# Identification and characterization of a novel, low-temperature-active GH8 endo-β-1,4-glucanase exhibiting broad pH stability from Antarctic *Glacieibacterium* sp. PAMC 29367

**DOI:** 10.3389/fmicb.2025.1682092

**Published:** 2025-10-21

**Authors:** Do Young Kim, Yung Mi Lee, Jong Suk Lee, Hyangmi Kim, Chung-Wook Chung

**Affiliations:** ^1^Microbiome Convergence Research Center, Korea Research Institute of Bioscience and Biotechnology (KRIBB), Daejeon, Republic of Korea; ^2^Division of Life Sciences, Korea Polar Research Institute, Incheon, Republic of Korea; ^3^Department of Bio Industry, Gyeonggido Business & Science Accelerator (GBSA), Suwon, Republic of Korea; ^4^Department of Biological Resources Research, Nakdonggang National Institute of Biological Resources, Sangju, Republic of Korea; ^5^Department of Biological Sciences, Gyeongkuk National University, Andong, Republic of Korea

**Keywords:** GH8, endo-β-1, 4-glucanase, cold-adapted enzyme, broad pH stability, Antarctic, *Glacieibacterium* sp.

## Abstract

Endo-β-1,4-glucanase plays an essential role in the breakdown of cellulosic substances that consist of D-glucose units linked by β-1,4-glycosidic bonds. In this work, the gene encoding a novel extracellular glycoside hydrolase (GH) family 8 endo-β-1,4-glucanase (GluS) from *Glacieibacterium* sp. PAMC 29367, an Antarctic lichen (*Megaspora verrucosa*)-associated bacterial species, was identified, cloned, and characterized. The GluS gene (1080-bp) was predicted to express a non-modular endo-β-1,4-glucanase (38,347 Da) that possesses a single catalytic GH8 domain, showing 65.5% amino acid sequence identity with an uncharacterized endoglucanase from *Alphaproteobacteria bacterium* (GenBank accession number: PZN92894). Recombinant endo-β-1,4-glucanase proteins (rGluS: 39.0 kDa) produced in *Escherichia coli* BL21 exhibited the highest carboxymethylcellulose (CMC)-degrading activity at pH 5.0 and 40°C, while maintaining over 80% of maximal endo-β-1,4-glucanase activity even at 25°C. Furthermore, the enzyme exhibited notable stability across a broad pH range from 4.5 to 10.0. rGluS activity was greatly stimulated by >1.3-fold in the presence of 1 mM Co^2+^, whereas it was nearly completely inhibited by 0.5% sodium dodecyl sulfate or 5 mM *N*-bromosuccinimide. The specific activity (31.1 U mg^–1^) and *k*_*cat*_/*K*_*m*_ (11.02 mg^–1^ s^–1^ mL) values of rGluS for CMC were marginally greater than those for barley β-1,3-1,4-glucan, with a specific activity of 28.9 U mg^–1^ and *k*_*cat*_/*K*_*m*_ of 8.79 mg^–1^ s^–1^ mL for barley β-1,3-1,4-glucan. The recombinant enzyme demonstrated no detectable biocatalytic activity for *p*-nitrophenylglucopyranoside, *p*-nitrophenylcellobioside, D-cellobiose, and D-cellotriose, while it could cleave D-cellotetraose to generate two molecules of D-cellobiose. Moreover, rGluS-mediated degradation of D-cellopentaose led mainly to D-cellobiose production along with D-glucose and D-cellotriose, while its hydrolysis of CMC yielded D-cellotriose as the dominant end product, accompanied by D-glucose, D-cellobiose and D-cellotetraose. The substrate preferences and degradation profiles of rGluS on cellulosic materials supported its classification as a true GH8 endo-acting β-1,4-glucanase without transglycosylation activity. The findings of this study suggest that rGluS represents a novel, highly active, cold-adapted GH8 endo-β-1,4-glucanase exhibiting broad pH stability, and may serve as an effective candidate for low-temperature processing in the food and textile industries.

## 1 Introduction

Lignocellulosic biomass, which is predominantly composed of cellulose, hemicellulose, and lignin, is the most plentiful raw material available in nature (Akbarian et al., 2022). In plant biomass, the proportions of cellulose, hemicellulose, and lignin are typically estimated at 40–50, 25–35, and 16–33 wt%, respectively (Cai et al., 2017). Among these components, cellulose is a recyclable recalcitrant biomaterial comprising D-glucose molecules connected by β-1,4-glycosidic linkages along its main chain. The fibrous nature of the polysaccharide chains is essential to the structural integrity of plant cell walls due to their extensive interconnections with lignin and different hemicelluloses such as xylan, mannan, and arabinan (Horn et al., 2012).

Cellulolytic bacterial and fungal strains are ubiquitous on earth, inhabiting both mild and extreme environments such as fresh and sea water (de Menezes et al., 2008; Arneen et al., 2014), soda lakes (van Solingen et al., 2001), hot springs (Miroshnichenko et al., 2008), soil (López-Mondéjar et al., 2016), deep-sea sediment (Zeng et al., 2006), compost (Lv and Yu, 2013), the digestive tracts of vertebrates and invertebrates (Ueda et al., 2014; Handique et al., 2017), and polar regions (Duncan et al., 2008; Zhao et al., 2019). Consequently, to achieve complete biodegradation of rigid cellulose fibrils within these environments, microbes produce three classes of glycoside hydrolase (GH) enzymes that display specific substrate affinities. Of the known eleven types of β-glucanases with different EC numbers (Jin et al., 2023), endo-β-1,4-glucanase (EC 3.2.1.4), cellobiohydrolase (EC 3.2.1.91), and β-glucosidase (EC 3.2.1.21) exemplify such cellulolytic enzymes, which cooperatively deconstruct cellulose fibrils into D-glucose molecules (Dimarogona et al., 2013; Bhardwaj et al., 2021; Sutaoney et al., 2024). Among these biocatalysts, endo-β-1,4-glucanases, which play a central role in cellulose deconstruction, are presently classified as six types of retaining enzymes assigned to GH families 5, 7, 10, 12, 16, 44, and 51, and seven types of inverting enzymes in GH families 6, 8, 9, 45, 48, 74, and 124, based on sequence similarity within their structurally-related catalytic domains (Liu et al., 2021)^1^.

Compared to mesophilic and thermophilic enzymes, cold-adapted biocatalysts exhibiting superior enzymatic activity at temperatures below 25°C may offer increased bio-economic viability. This is because cold-adapted enzyme-mediated low-temperature processes in bioindustry provide important benefits, as they eliminate the need for thermal treatments that may compromise product quality, cost-effectiveness, and sustainability in commercial production (Santiago et al., 2016). As a result, cold-adapted endo-β-1,4-glucanases with enhanced biocatalytic efficiency have attracted considerable interest as promising candidates for various bioindustrial applications, particularly in food and textile processes (Vester et al., 2014; Al-Ghanayem and Joseph, 2020). Currently, several cold-active or cold-adapted β-1,4-glucan-hydrolyzing enzymes showing distinctive molecular and biochemical characteristics, from diverse GH families, have been isolated and functionally characterized from marine invertebrates and microorganisms inhabiting cold environments (Yang and Dang, 2011; Bhat et al., 2013; Song et al., 2017; Zhao et al., 2019; Kim et al., 2022). However, so far, a β-1,4-glucan-degrading enzyme (rGluL) from Antarctic *Lichenicola cladoniae* PAMC 26568 remains the only cold-adapted endo-β-1,4-glucanase from GH family 8 identified and enzymatically characterized from polar microorganisms (Kim et al., 2022). Therefore, the biocatalytic and structural characteristics of cold-adapted GH8 endo-β-1,4-glucanases produced by polar microorganisms are not well documented. This emphasizes the need for further exploration into low-temperature-active GH8 endo-β-1,4-glucanases from Arctic and Antarctic microbes to discover industrially valuable biocatalysts with desirable functional attributes.

Lichens, which are classically defined as a mutualistic relationship between fungi and algae, have also been found to possess a diversity of internal lichen-associated bacteria (Bates et al., 2011). Consistent with this, our recent findings clearly indicated that various Antarctic lichens contained psychrophilic bacterial communities integral to the lichen symbiosis (Noh et al., 2021). Accordingly, to identify highly active cold-adapted cellulose-degrading biocatalysts, we performed an *in silico* analysis of the complete genome sequence of *Glacieibacterium* sp. PAMC 29367 [formerly *Polymorphobacter* sp. PAMC 29367 (Noh et al., 2021)], which was isolated from a lichen specimen of *Megaspora verrucose* (Ach.) Hafellner & V. Wirth, collected at Barton Peninsula, King George Island, Antarctica. The present study reports the molecular and functional characteristics of a newly identified, low-temperature-active GH8 endo-β-1,4-glucanase with broad pH stability derived from *Glacieibacterium* sp. PAMC 26367. The ecological significance of cellulolytic polar microorganisms regarding the bioremediation of lignocellulosic wastes in the Antarctic environment is also described.

## 2 Materials and methods

### 2.1 Carbon substrates

Oligomeric and polymeric substrates derived from D-glucose, such as D-cellobiose (C_2_), D-cellotriose (C_3_), D-cellotetraose (C_4_), D-cellopentaose (C_5_), D-cellohexaose (C_6_), barley β-1,3-1,4-glucan (low viscosity), and curdlan used in this study were supplied by Megazyme International Ireland Ltd., (Wicklow, Ireland). In contrast, *p*-nitrophenyl (PNP)-sugar derivatives (PNP-glucopyranoside and PNP-cellobioside), D-glucose (C_1_), and other polymeric substrates such as Avicel PH-101, locust bean gum, chitosan, sodium carboxymethylcellulose (CMC), and beechwood xylan were purchased from Sigma-Aldrich (St. Louis, MO, USA).

### 2.2 Cloning of the endo-β-1,4-glucanase gene

For the preparation of *Glacieibacterium* sp. PAMC 29367 genomic DNA, the strain was grown aerobically in R2A agar (BD Difco, Franklin Lakes, NJ, USA) for 14 days and 10°C, and the genomic DNA was then extracted from the cells collected by centrifugation using a Mini Tissue DNA kit (Cosmo Genetech Co., Ltd., Seoul, Korea), in accordance with the manufacturer’s instructions (Noh et al., 2021). The purified DNA served as a template for polymerase chain reaction (PCR) amplification of the gene encoding the mature GluS proteins, which was performed with a T100TM thermal cycler (Bio-Rad Laboratories, Inc., Seoul, Korea). Two gene-specific oligonucleotides containing the restriction sites for *Nde*I and *Hin*dIII were synthesized as follows: GluS-F (5′-*CATATG*TGTGCCAAGGCAAGCGG-3′) and GluS-R (5′-AAGCTTCTAGATTTGCGTCAGCAGCGC-3′). The PCR mixture (50 μL) included 2.5 U of FastStart Taq DNA polymerase (Roche, Basel, Switzerland), 250 μM of each dNTP, 2 pmol of each oligonucleotide, 20 ng of template DNA, and a PCR buffer. The thermal cycling conditions consisted of initial denaturation at 95 °C for 4 min, followed by 30 cycles of 30 s at 95 °C, 30 s at 64 °C, and 1 min at 72 °C. Following PCR, the amplified gene fragments were electrophoretically separated on a 1.2% agarose gel and subsequently purified from the excised gel bands containing the 1035-bp target gene fragments with a NucleoSpin Gel and PCR Clean-up kit (Macherey-Nagel, Düren, Germany). The obtained gene fragments were incorporated by ligation into a pGEM-T easy vector (Promega, Madison, WI, USA) for 3 h at 16 °C, followed by transformation of the ligation mixture into *Escherichia coli* DH5α competent cells. The constructed pGEM-T easy/*gluS* vectors were extracted using a NucleoSpin Plasmid (Macherey-Nagel) from recombinant cells cultivated in 50 mL of ampicillin (100 mg/L)-containing Luria-Bertani (LB) broth (BD Difco, Franklin Lakes, NJ, USA) at 180 rpm and 37°C for 12 h. Next, the recombinant vectors were cleaved with restriction endonucleases *Nde*I and *Hin*dIII to produce the *gluS* fragments with compatible ends. The resulting gene fragments, after a further purification using a NucleoSpin Gel and PCR Clean-up (Macherey-Nagel), were ligated into a pET-28a(+) expression vector (Novagen, Darmstadt, Germany) with matching sticky ends, and the resulting pET-28a(+)/*gluS* constructs were transformed into *E. coli* BL21.

### 2.3 Overproduction and isolation of recombinant endo-β-1,4-glucanase proteins

Using a 5-L baffled flask containing LB broth (1 L) supplemented with kanamycin (25 mg/L), recombinant endo-β-1,4-glucanase (rGluS) with an N-terminal six-histidine [(His)_6_] tag was produced by culturing recombinant *E. coli* BL21 cells harboring pET-28a(+)/gluS. The culture was maintained in a rotary shaker at 150 rpm for 16 h at 28 °C. Induction of rGluS overexpression was initiated by adding 1.0 mM isopropyl β-D-1-thiogalactopyranoside (IPTG) once the optical density at 600 nm reached approximately 0.5. Upon completion of cultivation, the rGluS-producing cells were harvested by centrifugation at 8000 × *g* for 20 min at 4 °C, then frozen at −20 °C for 3 h. For isolation of soluble rGluS proteins exhibiting high endo-β-1,4-glucanase activity, the recombinant *E. coli* BL21 cell pellet was homogeneously suspended in a binding buffer (pH 7.4) consisting of 20 mM sodium phosphate, 0.5 M NaCl, and 20 mM imidazole, followed by ultrasonic-mediated disruption of the cells. The soluble part with CMC-hydrolyzing activity, was collected by centrifugation at 15,000 × *g* for 20 min at 4 °C. The purification of the aforementioned (His)_6_-tagged rGluS proteins was simply achieved by affinity chromatography using a HisTrap HP (Cytiva, Uppsala, Sweden) (5.0 mL) column mounted on a fast protein liquid chromatography system (Amersham Pharmacia Biotech, Uppsala, Sweden), in accordance with the instruction provided by manufacturer. Elution of the N-terminal (His)_6_-tagged rGluS proteins from the column was performed by employing a linear imidazole gradient (20–500 mM) at a flow rate of 2.0 mL/min, following the recommended protocol. Fractions demonstrating high CMC-hydrolyzing activity were then pooled and desalted using a HiPrep 26/10 desalting column (Cytiva) with 50 mM sodium phosphate buffer (pH 6.0) as the mobile phase. The endo-β-1,4-glucanase-active fractions were combined and held in ice water for downstream analysis.

### 2.4 Protein analysis

The relative molecular mass of denatured rGluS proteins was determined by sodium dodecyl sulfate-polyacrylamide gel electrophoresis (SDS-PAGE) using a 12.0% gel. Following electrophoresis, staining of the gel with a 0.05% Coomassie Brilliant Blue R-250 solution (Bio-Rad Laboratories, Inc., Seoul, Korea) was done for 3 h to visualize distinct protein bands. The amount of protein in a sample was quantitatively determined with the Bio-Rad Protein Assay Dye Reagent Concentrate (Bio-Rad Laboratories, Inc.), using bovine serum albumin as a standard.

### 2.5 Enzyme assays

The endo-β-1,4-glucanase activity of rGluS was quantified by assaying the release of reducing sugars from the enzymatic hydrolysis of CMC using the 3,5-dinitrosalicylic acid (DNS) reagent. For this purpose, a standard D-glucose calibration curve was generated by plotting mean absorbance values against known concentrations, and this curve was subsequently used for the measurement of reducing sugars. The standard reaction mixture (0.5 mL) for the endo-β-1,4-glucanase assay was made up of 1.0% CMC and rGluS solution (0.05 mL) diluted in 50 mM sodium acetate buffer (pH 5.0). Enzyme assays were typically performed at 40 °C for 10 min, after which the enzyme reactions were promptly stopped by the addition of DNS reagent (0.75 mL) to the assay mixture. Thereafter, the colorimetric analysis was conducted by determining the absorbance at 540 nm, detecting the red-brown color developed after boiling the mixture for 5 min. One unit (U) of endo-β-1,4-glucanase activity toward CMC or barley β-1,3-1,4-glucan was defined as the quantity of rGluS necessary to liberate 1 μmol of reducing sugar per min under standard assay conditions.

### 2.6 Effects of pH, temperature, and chemicals on the endo-β-1,4-glucanase activity

The influence of pH on the degradation activity of rGluS against CMC was explored by reacting the enzyme with the substrate across pH values from 4.0 to 10.5 using the following buffer systems (each 50 mM) at 40 °C for 10 min: sodium acetate (pH 4.0–5.5), sodium phosphate (pH 5.5–7.5), Tris-HCl (pH 7.5–9.5), and glycine-NaOH (pH 9.5–10.5). However, the pH stability of rGluS in each buffer was examined by measuring its residual β-1,4-glucan-degrading activity after completing the biocatalytic reaction carried out at 40 °C for 10 min. For this assay, the degradation reaction was started by inserting 1% CMC to the reaction mixture immediately after preincubating rGluS at 3 °C for 1 h in the respective pH buffers without the substrate present. The effect of temperature on the biocatalytic activity of rGluS for CMC hydrolysis was investigated by incubating the enzyme with the substrate at 1 °C, 5 °C, 10 °C, 15 °C, 20 °C, 25 °C, 30 °C, 35 °C, 40 °C, 45 °C, 50 °C, 55 °C, and 60 °C for 10 min in 50 mM sodium acetate buffer (pH 5.0). In parallel, the thermostability of rGluS at temperatures of 3 °C, 10 °C, 18 °C, 25 °C, 30 °C, 35 °C, 40 °C, 45 °C, and 50 °C was determined by assaying its residual β-1,4-glucan-degrading activity after terminating the enzyme reaction that was fulfilled for 10 min in 50 mM sodium acetate buffer (pH 5.0). In this thermostability test, the enzyme was preincubated at the designated reaction temperature in the absence of CMC for 1 h at pH 5.0, followed by initiating the biocatalytic reaction by introducing the substrate (1%) into the assay mixture. The impact of divalent cations (each 1 mM) and various chemical compounds (each 5 mM or 0.5%) on the β-1,4-glucan-degrading activity of rGluS was evaluated after preincubating the enzyme at 3 °C for 10 min in a reaction mixture containing 1% CMC and the chemical of concern.

### 2.7 Analysis of the degradation products

To evaluate the degradation profiles of D-cellooligomers (C_2_–C_6_, each 1 mg) and CMC (2 mg) by rGluS (10 μg), the biocatalytic reactions were performed at 35 °C for 6 h in 50 mM sodium acetate buffer (pH 5.0), during which the enzyme retained over 90% of its initial β-1,4-glucan-degrading activity. The reactions were terminated by exposing the reaction mixtures at 100 °C for 5 min. Next, the resulting degradation products derived from the cellulosic substrates were analyzed by liquid chromatography mass spectrometry (LC-MS) with C_1_ and D-cellooligomers (C_2_–C_6_) as reference standards. Quantitative evaluation of the degradation products was accomplished employing ultra high performance liquid chromatography (UHPLC) and a Vanquish UHPLC system (Thermo Fisher Scientific Inc., Waltham, MA, USA) equipped with an ACQUITY BEH Amide column (1.7 μm, 2.1 mm × 100 mm, Waters Corp., Milford, MA, USA) and Orbitrap Fusion (Thermo Electron Co., Waltham, MA, USA). Elution of the degradation products from the column was performed using a mobile phase comprising water with 0.1% NH_4_OH (solvent A) and acetonitrile with 0.1% NH_4_OH (solvent B), at a flow rate of 0.4 mL/min. The solvent gradient was as follows: 85% solvent B at 0–3 min, 60% solvent B at 10 min, and 40% solvent B at 10.1–12 min. MS detection was performed in negative ion mode over the scan range m/z 140–1400.

## 3 Results and discussion

### 3.1 Molecular characterization of the GH8 endo-β-1,4-glucanase gene

**FIGURE 1:**
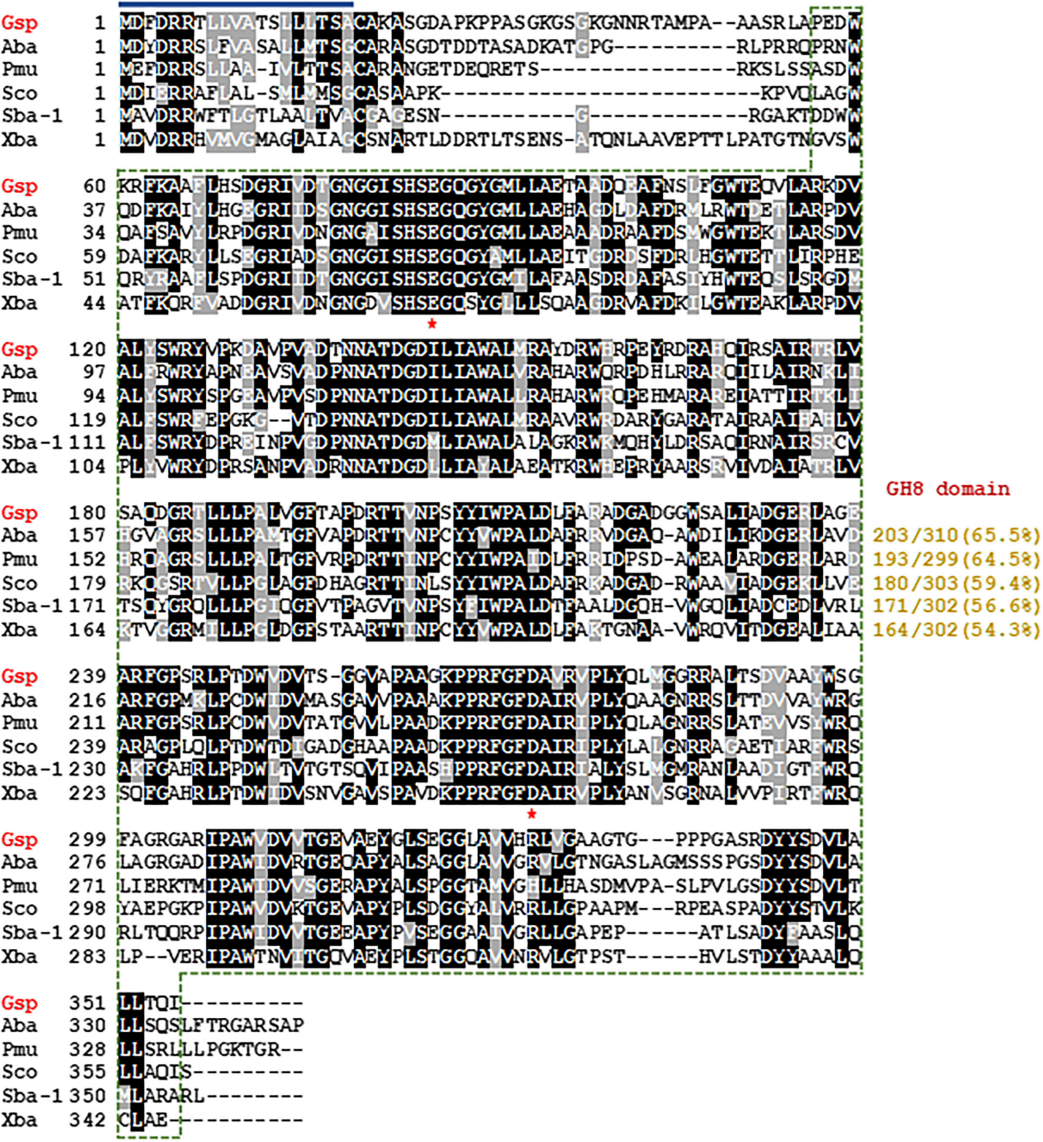
Structure-based sequence alignment of *Glacieibacterium* sp. PAMC 29367 GH8 endo-β-1,4-glucanase and its GH8 functional analogues. Shown are sequences (GenBank accession numbers) of *Glacieibacterium* sp. (Gsp) PAMC 29367 endo-β-1,4-glucanase (PV751198), *Alphaproteobacteria bacterium* (Aba) endoglucanase (PZN92894), *Polymorphobacter multimanifer* (Pmu) GH8 protein (WP_184199733), *Sphingomonas colocasiae* (Sco) endoglucanase (MBY8824380), *Sphingomonadales bacterium* (Sba-1) 63-6 endoglucanase (OJW72739), and *Xanthomonadaceae bacterium* (Xba) endoglucanase (RYD28981). The identical and similar amino acids are exhibited by black and gray boxes, respectively. The predicted signal peptide is indicated by a dark blue bar and GH8 domain is outlined by a green dotted line. Strictly conserved amino acid residues (Glu83 and Glu271), which participate in biocatalysis, are marked with red asterisks.

The extracellular GH8 endo-β-1,4-glucanase (GluS) gene (1080-bp) of an Antarctic lichen (*M. verrucose*)-associated bacterium, *Glacieibacterium* sp. PAMC 29367, was identified through an *in silico* analysis of its whole genome sequence and was deposited in GenBank nucleotide sequence database under accession number PV751198. According to assessments using the Compute pI/MW tool,^2^ the GluS gene was predicted to code for a premature protein (359 amino acids) with a deduced molecular mass of 38,347 Da and a theoretical isoelectric point (pI) of 7.83 (Figure 1). Moreover, in premature GluS, its signal peptide cleavage site was estimated to be likely between Ala19 and Cys20 in the N-terminal region, as examined by the SignalP 6.0 server^3^. In contrast, the mature form of GluS lacking the signal peptide was assessed to be a basic protein consisting of 340 amino acids, with a deduced molecular mass of 36,241 Da and a theoretical pI of 7.99. Detailed Protein BLAST and Pfam analyses demonstrated that the premature GluS was a non-modular endo-β-1,4-glucanase made up of a single catalytic GH8 domain (from Pro55 to Ile359) without any additional substrate-binding domains (Figure 1). Analogous to GluS, most other well-characterized GH8 enzymes have also been reported as non-modular endo-β-1,4-glucanases comprising only a single catalytic GH8 domain (Bhat et al., 2013; Chen et al., 2020; de Melo et al., 2021; Kim et al., 2022). On the other hand, certain GH8 endo-β-1,4-glucanases from *Acetivibrio thermocellus* ATCC 27405 (formerly *Clostridium thermocellum*) (Bélguin et al., 1985) and *Ruminococcus champanellensis* (Moraïs et al., 2016) have been described as bi-modular enzymes consisting of an N-terminal catalytic GH8 domain as well as a C-terminal dockerin_1 domain.

**FIGURE 2:**
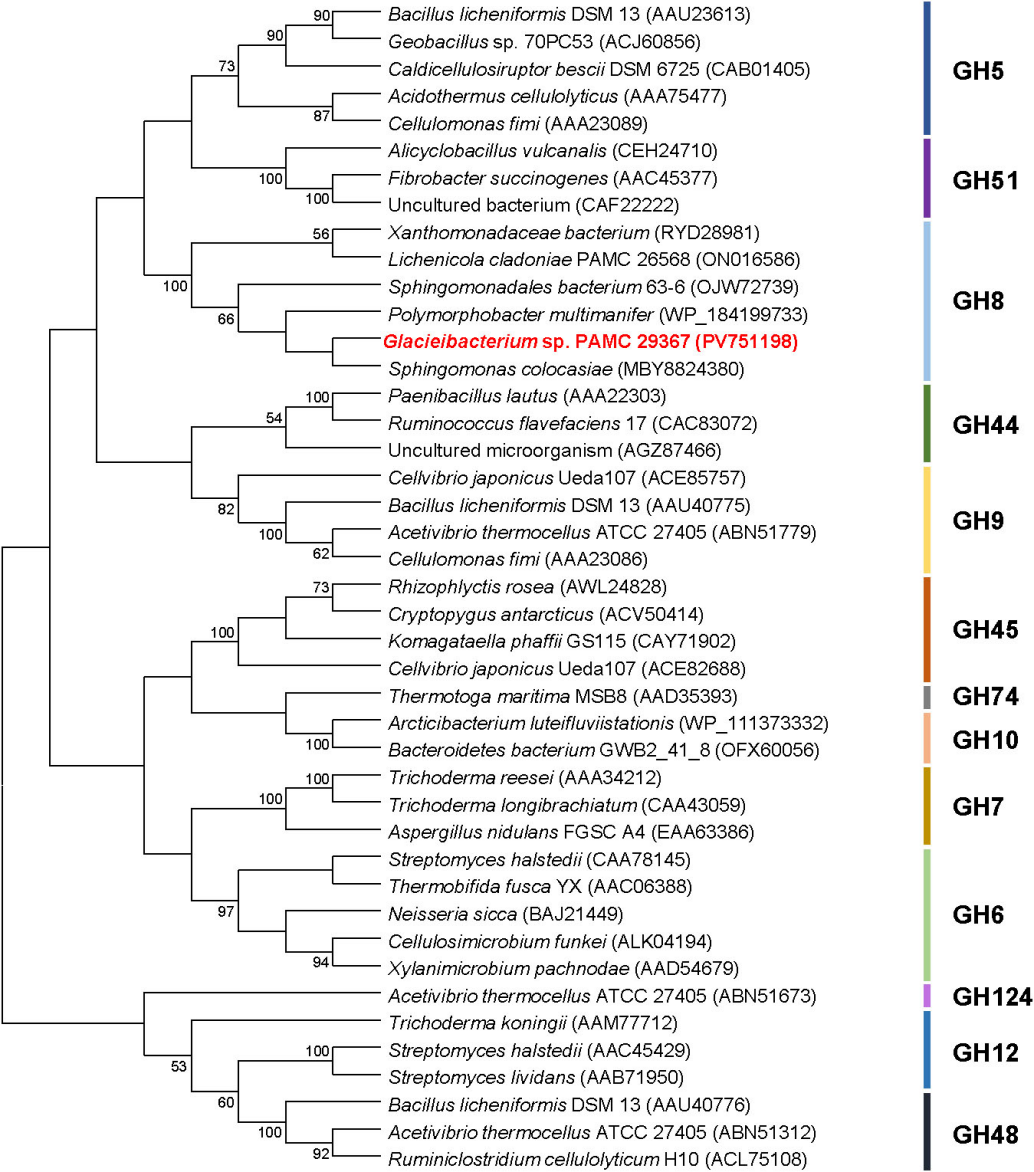
Phylogenetic analysis of *Glacieibacterium* sp. PAMC 29367 GH8 endo-β-1,4-glucanase (GluS) and its closely related functional analogues. Multiple sequence alignment of proteins was carried out using ClustalW in the MEGA11 software (Tamura et al., 2021). The amino acid sequence data used for phylogenetic analysis were retrieved from the GenBank database (Supplementary material).

According to the Carbohydrate-Active enZYmes (CAZy) database,^4^ GH family 8 includes a range of structurally-related carbohydrolases with specific biological function(s), such as endo-β-1,4-glucanase (EC 3.2.1.4), endo-β-1,3(4)-glucanase (EC 3.2.1.6), endo-β-1,3-1,4-glucanase (EC 3.2.1.73), reducing-end-xylose releasing exo-oligoxylanase (EC 3.2.1.156), endo-β-1,3-xylanase (EC 3.2.1.32), endo-β-1,4-xylanase (EC 3.2.1.8), and chitosanase (3.2.1.132)^5^. Consequently, phylogenetic analysis was performed to clarify the evolutionary relationship between GluS and its functional analogues. The phylogenetic tree demonstrated that the amino acid sequence of GluS shared a close evolutionary relationship with that of other GH8 endo-β-1,4-glucanases (Figure 2). Multiple sequence alignment between GluS and its structural homologs further proved that the catalytic GH8 domain of GluS was most similar to that of an uncharacterized *Alphaproteobacteria bacterium* endoglucanase (GenBank accession number: PZN92894), showing a sequence identity of 65.5% as reported in the National Center for Biotechnology Information (NCBI) database (Figure 1). In addition, a protein BLAST search indicated that the catalytic GH8 domain of GluS shared 64.5%, 59.4%, 56.6%, and 54.3% sequence identity with that of *Polymorphobacter multimanifer* GH8 protein, *Sphingomonas colocasiae* endoglucanase, *Sphingomonadales bacterium* endoglucanase, and *Xanthomonadaceae bacterium* endoglucanase, respectively, none of which have been experimentally characterized to date. Collectively, the relatively low sequence identity (<66.0%) between the catalytic GH8 domain of GluS and its structural homologs strongly implied that it might be a novel GH8 endo-β-1,4-glucanase with unique biocatalytic features. Two strictly conserved residues in the active site of GluS, Glu85 as the proton donor and Asp271 as the acceptor, were consistent with those identified in the active site of other GH8 endo-β-1,4-glucanases (Bhat et al., 2013; Cano-Ramírez et al., 2016; Huang et al., 2021; Kim et al., 2022; Figure 1). However, it is noteworthy that a cold-adapted GH8 endo-β-1,4-glucanase (BpEG) from *Burkholderia pyrrocinia* JK-SH007 has been reported to utilize two catalytic Glu83 (proton donor) and Glu271 (acceptor) residues during biocatalysis (Chen et al., 2020).

### 3.2 Overexpression and purification of recombinant endo-β-1,4-glucanase

Owing to their high hydrophobicity, certain non-modular and modular endo-β-1,4-glucanases from various GH families have been reported to be produced in *E. coli* BL21 primarily as inactive protein aggregates (Kim et al., 2016; Wierzbicka-Woś et al., 2019), which can subsequently be transformed to their active form via an on-column refolding approach (Singh et al., 2015). Likewise, rGluS bearing an N-terminal (His)_6_-tag was mostly produced as substantial amounts of inactive inclusion bodies, accompanied by a minor fraction of water-soluble active protein, when overexpressed in recombinant *E. coli* BL21 carrying pET-28a(+)/*gluS*. It is interesting to note that a significant proportion of the non-modular GH8 rGluS proteins was produced in an inactive state because other cold-adapted GH8 endo-β-1,4-glucanases were demonstrated to be solely produced in their active form (Zhang et al., 2013; Chen et al., 2020; de Melo et al., 2021; Kim et al., 2022). In this study, the biocatalytic features of rGluS were characterized using its native, highly active endo-β-1,4-glucanase form that was easily purified from the soluble cell lysate via affinity column chromatography without necessitating an on-column refolding procedure.

**FIGURE 3:**
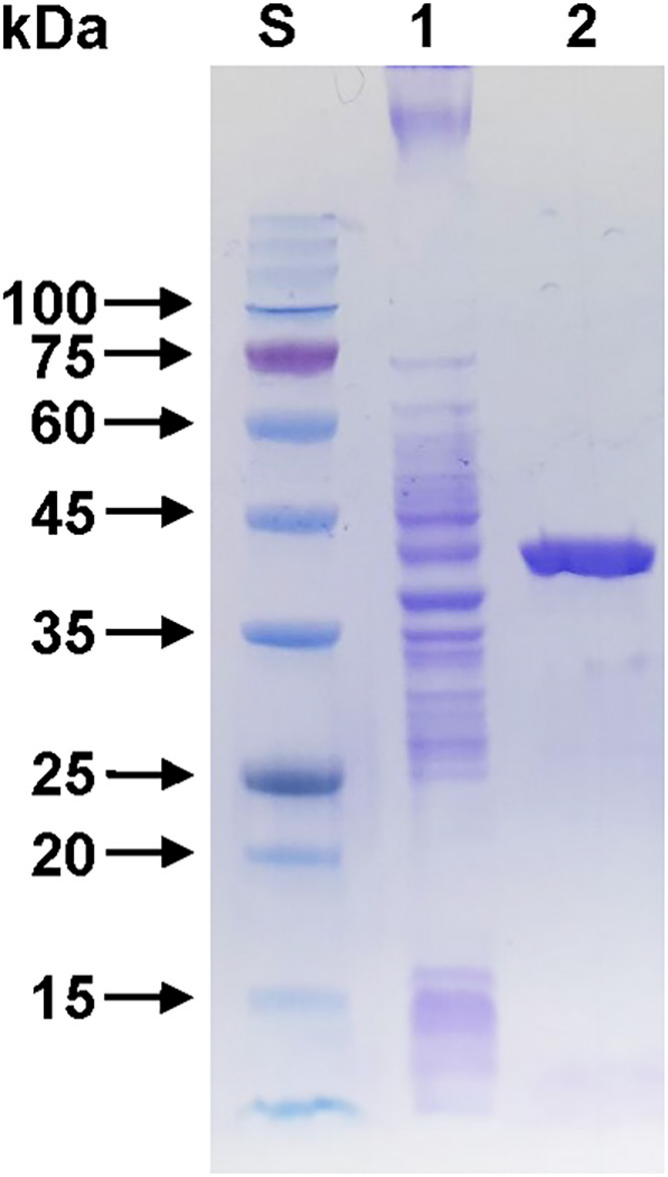
Sodium dodecyl sulfate-polyacrylamide gel electrophoresis (SDS-PAGE) of the purified rGluS after affinity chromatography on HisTrap HP. Lane S, standard marker proteins; lane 1, the soluble cell lysate of rGluS-expressing *E. coli* BL21 after IPTG induction; lane 2, purified rGluS.

Sodium dodecyl sulfate-polyacrylamide gel electrophoresis analysis revealed that the relative molecular mass of (His)_6_-tagged rGluS was approximately 39.0 kDa (Figure 3), which corresponded well with its predicted molecular mass (38,451 Da) obtained from the Compute pI/MW tool^6^. The observed molecular size (39.0 kDa) of rGluS was closely aligned with that reported for other GH8 endo-β-1,4-glucanases, which range from 38.0 to 40.4 kDa and are derived from cold desert soil in Ladakh (Bhat et al., 2013), *L. cladoniae* PAMC 26568 (Kim et al., 2022), *Paenibacillus* sp. YD236 (Dong et al., 2016), and *Bursaphelenchus xylophilus* (Zhang et al., 2013; Table 1). Furthermore, rGluS (39.0 kDa) also closely resembled the molecular size of a thermostable GH8 endo-β-1,4-glucanase (GH8ErCel: 38.0 kDa) from *Enterobacter* sp. R1 (Ontañon et al., 2019). By contrast, previous studies have indicated that a mesophilic GH8 endo-β-1,4-glucanase (celA1805) (53.0 kDa) from *Bacillus subtilis* B111 (Huang et al., 2021) and the cold-adapted GH8 BpEG (60.0 kDa) (Chen et al., 2020) display greater molecular sizes than rGluS (39.0 kDa).

**TABLE 1:** Enzymatic features of various bacterial GH8 endo-β-1,4-glucanases.

Source	Enzyme	M*_*r*_* (kDa)	Opt. pH	Opt. temp. (*^o^*C)	Specific activity (U mg^–1^)	References
*Glacieibacterium* sp. PAMC 29367	rGluS	39.0	5.0	40	28.9^a^, 31.1^b^	This study
*Lichenicola cladoniae* PAMC 26568	rGluL	38.0	4.0	45	15.1^a^, 8.3^b^	Kim et al., 2022
*Xanthomonas citri* subsp. citri	*Xac*Cel8	41.5	5.5-7.5	40	16.6^a^, 12.7^b^	de Melo et al., 2021
*Burkholderia pyrrocinia* JK-SH007	BpEG	60.0	6.0	35	11.2^b^	Chen et al., 2020
*Paenibacillus* sp. YD236	PgluE8	40.4	5.5	50	11.0^a^, 2.3^b^	Dong et al., 2016
Cold desert soil in Ladakh	CEL8M	39.0	4.5	28	4.7^b^	Bhat et al., 2013
*Enterobacter* sp. R1	GH8ErCel	38.0	7.0	60	49.8^a^, 4.1^b^	Ontañon et al., 2019
*Bacillus subtilis* B111	celA1805	53.0	6.0	50	12.8^a^, 7.6^b^	Huang et al., 2021
*Bacillus circulans* KSM-N257	Egl-257	43.0	8.5	55	17.7^b^	Hakadama et al., 2002
*Bursaphelenchus xylophilus*	Cen219	40.0	6.0	50	189.6^a^, 107.2^b^	Zhang et al., 2013
*Serratia proteamaculans* CDBB-1961	Cel8A	41.0	7.0	40	0.8^b^	Cano-Ramírez et al., 2016
*Halomonas* sp. S66-4	Cel8H	36.0	5.0	45	1.9^a^, 4.9^b^	Huang et al., 2010

^a^Specific enzyme activity for barley β-1,3-1,4-glucan.

^b^Specific enzyme activity for CMC.

### 3.3 Enzymatic characterization of rGluS

**FIGURE 4:**
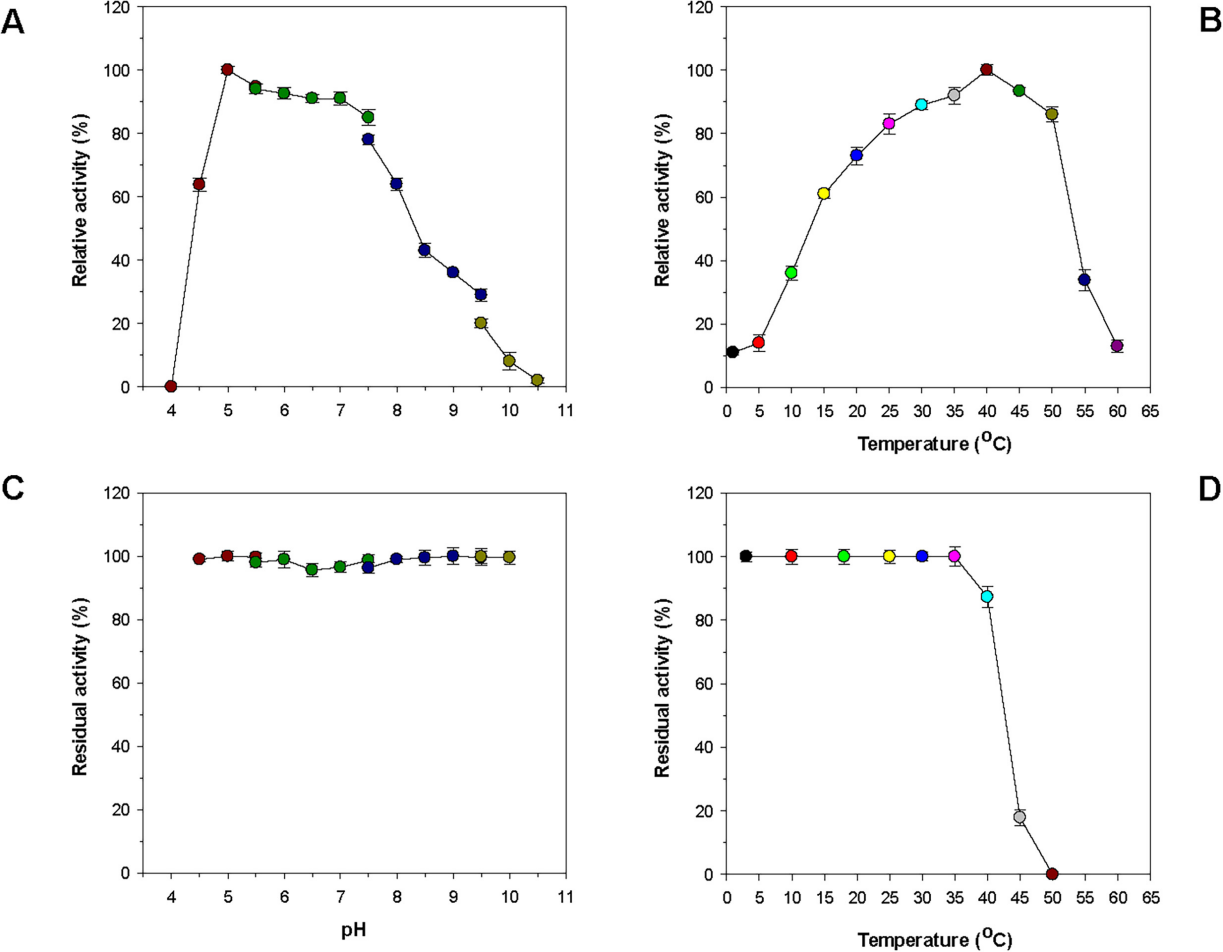
Effects of pH **(A)** and temperature **(B)** on the endo-β-1,4-glucanase activity of rGluS and effects of pH **(C)** and temperature **(D)** on the stability of rGluS. The optimum pH of rGluS was examined employing the following buffers at 50 mM: sodium acetate (pH 4.0–5.5), sodium phosphate (pH 5.5–7.5), Tris-HCl (pH 7.5–9.5), and glycine-NaOH (pH 9.5–10.5). The optimum temperature of rGluS was investigated at various temperatures (1 °C–60 °C) in 50 mM sodium acetate buffer (pH 5.0). The pH stability of rGluS was assessed by ascertaining the residual endo-β-1,4-glucanase activity after pre-incubation of the enzyme using the aforementioned buffer systems (50 mM) at 3 °C for 1 h. The thermostability of rGluS was evaluated by measuring the residual endo-β-1,4-glucanase activity after pre-incubation of the enzyme at 3 °C, 10 °C, 18 °C, 25 °C, 30 °C, 35 °C, 40 °C, 45 °C, and 50 °C in 50 mM sodium acetate buffer (pH 5.0) for 1 h. The values are mean ± SD of triplicate tests.

Table 1 reveals that many bacterial GH8 endo-β-1,4-glucanases most efficiently deconstructed barley β-1,3-1,4-glucan or CMC under weakly acidic to neutral pH conditions, specifically between 5.5 and 7.0. In contrast, it has also been demonstrated that a GH8 endo-β-1,4-glucanase (Egl-257) from *Bacillus circulans* KSM-N257 exhibits optimal barley β-1,3-1,4-glucan degradation at an alkaline pH of 8.5 (Hakadama et al., 2002). In this work, rGluS was observed to achieve maximal degradation of CMC when assayed at pH 5.0 and 40°C (Figures 4A, B). Moreover, the endo-β-1,4-glucanase activity of rGluS was markedly diminished at pH values below 5.0 and declined progressively within the pH range of 7.5–10.5. These findings supported the classification of this enzyme as an acidic GH8 endo-β-1,4-glucanase, displaying distinct characteristics compared to other functional homologs belonging to GH family 8 (Table 1). The optimal pH (5.0) for rGluS in CMC degradation matched that of *Halomonas* sp. S66-4 GH8 endo-β-1,4-glucanase (Cel8H) (Huang et al., 2010) but was higher than that of *L. cladoniae* PAMC 26568 GH8 endo-β-1,4-glucanase (rGluL), which showed its peak biocatalytic activity at pH 4.0 (Kim et al., 2022). However, the maximum degradation temperature (40°C) of rGluS for CMC was lower than that (45°C) of Cel8H (Huang et al., 2010) and that (45°C) of rGluL (Kim et al., 2022) for the same substrate. In a manner similar to rGluS, certain cold-adapted GH8 endo-β-1,4-glucanases have also been shown to achieve maximal deconstruction of β-1,4-glucan polysaccharides at temperatures below 40°C. Representative enzymes include a GH8 endo-β-1,4-glucanase (*Xac*Cel8) from *Xanthomonas citri* subsp. *Citri* (de Melo et al., 2021), a GH8 endo-β-1,4-glucanase (CEL8M) from cold desert soil in Ladakh (Bhat et al., 2013), and BpEG (Chen et al., 2020), which achieved the highest biocatalytic activity toward barley β-1,3-1,4-glucan at 40 °C, 35 °C, and 28°C, respectively. It is noteworthy that rGluS exhibited over 80% of its maximum biocatalytic activity even at the lower temperature of 25°C (Figure 4B). Additionally, the enzyme retained the capacity to hydrolyze CMC even at a cold temperature of 1°C, reaching approximately 12% of its maximum endo-β-1,4-glucanase activity, thus supporting its classification among cold-adapted GH8 endo-β-1,4-glucanases such as rGluL, *Xac*Cel8, BpEG, PgluE8, and CEL8M (Table 1). It is hypothesized that similar to rGluL (Kim et al., 2022), the adaptation of rGluS to cold environments may arise from enhanced structural flexibility that enables efficient biocatalysis at low temperatures (Parvizpour et al., 2015). Remarkably, rGluS appeared to exhibit substantial stability across a broad pH range (4.5–10.0) because the enzyme preserved > 95% of its residual biocatalytic activity even after pre-incubation of 1 h in the absence of CMC at these pH values (Figure 4C). It was also observed that rGluS was quite stable at temperatures below 35°C, but its thermostability decreased drastically when exposed to temperatures exceeding 40°C for 1 h in the absence of the substrate (Figure 4D). These results suggested that rGluS was a thermolabile, cold-adapted GH8 endo-β-1,4-glucanase similar to *Xac*Cel8 (de Melo et al., 2021), BpEG (Chen et al., 2020), and CEL8M (Bhat et al., 2013). Taken together, these data supported the potential application of cold-adapted rGluS displaying broad pH stability as a biocatalyst suitable for low-temperature processes in the bioindustry.

**FIGURE 5:**
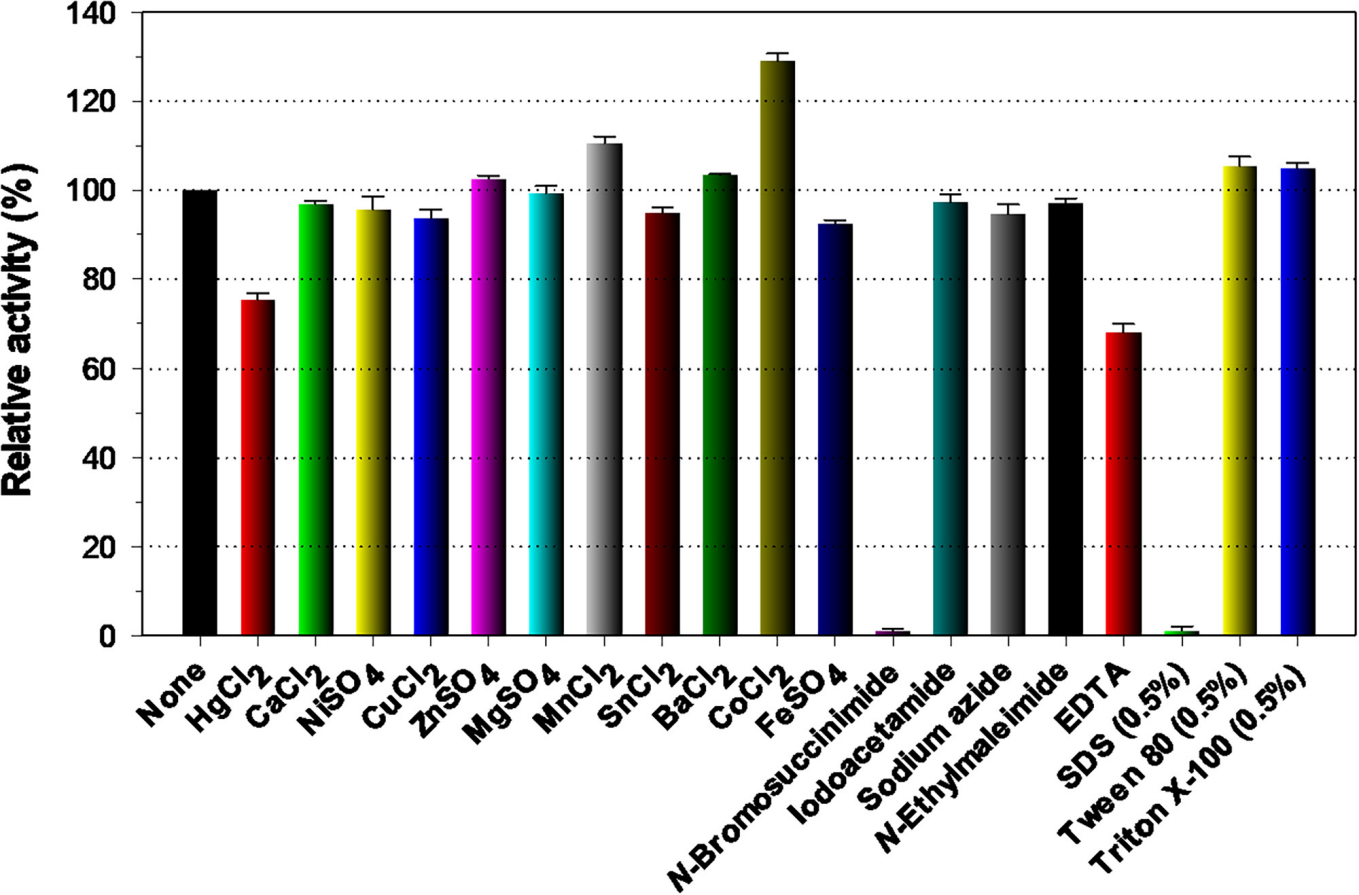
Effects of divalent cations (1 mM) and chemical reagents (5 mM) on the endo-β-1,4-glucanase activity of rGluS. The values are mean ± SD of triplicate tests.

Because various divalent cations including Co^2+^and Mn^2+^ ions are commonly present in plant biomass (Vassilev et al., 2012), it is noteworthy that the biocatalytic activity of rGluS could be greatly enhanced up to approximately 1.3-fold when preincubated in the presence of 1 mM Co^2+^ (Figure 5). The pronounced activation (1.3-fold) of rGluS by 1 mM Co^2+^ was compatible to the enhancement (approximately 1.8-fold) observed in a cold-adapted GH8 *Xac*Cel8 enzyme (de Melo et al., 2021) subjected to the same divalent cations (10 mM). However, it has been demonstrated that Co^2+^ ions exert minimal or no effect on the biocatalytic activity of Cel8H (Huang et al., 2010), Egl-257 (Hakadama et al., 2002), or a GH8 endo-β-1,4-glucanase (Cen219) from *B. xylophilus* (Zhang et al., 2013). A similar effect on the stimulation of rGluS activity was also observed when the enzyme assay was performed with 1 mM Mn^2+^. In this instance, the upregulation of its biocatalytic activity exerted by Mn^2+^ was shown to be 1.1-fold, while the biocatalytic activity of a GH8 endo-β-1,4-glucanase (Cel8A) from *Serratia proteamaculans* CDBB-1961 was previously reported to increase by about 1.5-fold in the presence of the identical divalent cations (1 mM) (Cano-Ramírez et al., 2016). Notably, when compared to the endo-β-1,4-glucanase activities of rGluS and Cel8A (Cano-Ramírez et al., 2016), the activities of *Xac*Cel8 (de Melo et al., 2021) and PgluE8 (Dong et al., 2016) were reported to be suppressed by >20% upon exposure to 10 mM Mn^2+^. It is also interesting to note that neither stimulatory nor inhibitory effects on rGluS activity were observed when 1 mM Ca^2+^, Ni^2+^, Zn^2+^, Mg^2+^, Cu^2+^, Sn^2+^, Ba^2+^ and Fe^2+^ were assessed (Figure 5). In contrast, a complete loss of the Cel8A activity by Zn^2+^ ions (Cano-Ramírez et al., 2016) and a substantial reduction (approximately 55%) of the Cel8H activity by Cu^2+^ ions (Huang et al., 2010) have been documented to date. Moreover, Fe^2+^ ions have been found to cause a remarkable downregulation (>90%) of rGluL activity (Kim et al., 2022) and a powerful upregulation (approximately 1.6-fold) of Cel8H activity (Huang et al., 2010). In this study, it was determined that sulfhydryl reagents (each 5 mM), such as iodoacetamide, sodium azide, and *N*-ethylmaleimide, as well as non-ionic surfactants (each 0.5%), including Tween 80 and Triton X-100, did not significantly influence rGluS activity. These observations were consistent with the fact that rGluL was almost insensitive to the same sulfhydryl reagents and non-ionic surfactants (Kim et al., 2022). Among the chemicals tested, it has been reported that Hg^2+^ and *N*-bromosuccinimide act as tryptophan (Trp) residue-directed modifiers oxidizing the indole ring of catalytic Trp residues in various GH enzymes, which are essential for enzyme-substrate interactions (Zolotnitsky et al., 2004). In fact, a strong enzyme inhibition by the two Trp residue-specific chemicals was also observed in some GH8 endo-β-1,4-glucanases, such as rGluL (Kim et al., 2022), Cel8H (Huang et al., 2010), and CEL8M (Bhat et al., 2013), when treated with the same reagents. However, Figure 5 shows that *N*-bromosuccinimide exhibited notable toxicity toward rGluS, while Hg^2+^ could only partially suppress rGluS activity by about 32%. The partial inhibition (32%) of rGluS by Hg^2+^ was similar to that (36%) of Cen219 (Zhang et al., 2013) and that (64%) of Cel8A (Cano-Ramírez et al., 2016) exposed to the same compound. Consistent with findings for other characterized GH8 endo-β-1,4-glucanases (Bhat et al., 2013; Zhang et al., 2013; Kim et al., 2022), SDS was a strong inhibitor that nearly abolished the biocatalytic activity of rGluS. The powerful inactivation of rGluS activity by SDS was comparable to the partial inhibition (50%) of the biocatalytic activity of *Bacillus subtilis* B111 GH8 endo-β-1,4-glucanase (celA1805) (Huang et al., 2021) by the same chemical. Meanwhile, similar to Cel8H (Huang et al., 2010), rGluS was partially downregulated by >30% of its original endo-β-1,4-glucanase activity after preincubation with the metal chelator EDTA in the absence of the CMC substrate. On the other hand, the positive or negative regulatory effect of 1 mM EDTA on the endo-β-1,4-glucanase activity of Cel8A (Cano-Ramírez et al., 2016) or rGluL (Kim et al., 2022) was found to be negligible.

### 3.4 Substrate specificity, kinetic parameters, and degradation products

So far, different GH8 endo-β-1,4-glucanases possessing peculiar biochemical properties have been identified from an environmental sample and diverse bacterial species (Table 1). Among these extracellular β-1,4-glucan-degrading enzymes, *Xac*Cel8 (de Melo et al., 2021), rGluL (Kim et al., 2022), BpEG (Chen et al., 2020), PgluE8 (Dong et al., 2016), and CEL8M (Bhat et al., 2013) represent cold-adapted GH8 endo-β-1,4-glucanases that have undergone both genetic and functional characterization. Conversely, GH8ErCel has been reported as a thermostable GH8 endo-β-1,4-glucanase exhibiting its maximum biocatalytic activity toward barley β-1,3-1,4-glucan at 60°C (Ontañon et al., 2019). It is important to also emphasize that rGluL (Kim et al., 2022), BpEG (Chen et al., 2020), GH8ErCel (Ontañon et al., 2019), Cel8H (Huang et al., 2010), *Xac*Cel8 (de Melo et al., 2021), and Egl-257 (Hakadama et al., 2002) are restricted in substrate specificity to cellulosic materials consisting of β-1,4-linked D-glucose, whereas celA1805 (Huang et al., 2021), Cen219 (Zhang et al., 2013), and Cel8A (Cano-Ramírez et al., 2016) represent bi-functional GH8 endo-β-1,4-glucanases capable of decomposing either chitosan or β-1,4-xylan together with β-1,4-glucans. Moreover, the biocatalytic activities of CEL8M (Bhat et al., 2013) and a GH8 endo-β-1,4-glucanase (Cel8Pa) from *Paenibacillus xylanivorans* A59 (Ghio et al., 2020) on three different polysaccharides, such as β-1,4-glucan, β-1,4-xylan, and chitosan, differed from those of the aforementioned true endo-β-1,4-glucanases. Accordingly, the substrate specificity of rGluS in this investigation was assessed employing PNP-sugar derivatives as well as cellulosic and hemicellulosic polysaccharides that possess unique microstructures (Table 2).

**TABLE 2:** Biocatalytic activity of rGluS for different substrates.

Substrate	Main linkage type	Specific activity (U mg^–1^)^a^	Relative activity (%)
Avicel PH-101	β-1,4	ND^b^	–
CMC	β-1,4	31.1 ± 0.1	100.0
Barley β-1,3-1,4-glucan	β-1,3 and β-1,4	28.9 ± 0.3	92.9
Curdlan	β-1,3	ND	–
Chitosan	β-1,4	ND	–
Locust bean gum	β-1,4	ND	–
Beechwood xylan	β-1,4	ND	–
PNP-glucopyranoside	β-1,4	ND	–
PNP-cellobioside	β-1,4	ND	–

^a^Specific activity was obtained from the three repeated experiments;

^b^Not detected.

It was found that rGluS could readily deconstruct CMC and barley β-1,3-1,4-glucan into low molecular weight products, with measured specific activities for CMC and barley β-1,3-1,4-glucan of 31.1 and 28.9 U mg^–1^, respectively. However, the enzyme failed to demonstrate biocatalytic activity toward polysaccharides lacking structural similarity as well as PNP-cellobioside and PNP-glucopyranoside. Additionally, unlike its action on amorphous CMC, rGluS did not induce the degradation of a crystalline β-1,4-glucan, Avicel PH-101. This observation is likely attributed to the high crystallinity of Avicel PH-101, which is thought to substantially limit enzymatic degradation rates (Hall et al., 2010). Taken together, these results confirmed that rGlus was a true, cold-adapted GH8 endo-β-1,4-glucanase that lacks additional carbohydrolase functions, closely resembling other GH8 functional analogues (Chen et al., 2020; de Melo et al., 2021; Kim et al., 2022). As listed in Table 1, to the best of our knowledge, rGluS currently exhibits the highest activity among cold-adapted GH8 endo-β-1,4-glucanases with a preference for hydrolyzing cellulosic substrates, surpassing previously characterized cold-adapted GH8 homologs (Bhat et al., 2013; Dong et al., 2016; Chen et al., 2020; de Melo et al., 2021; Kim et al., 2022). Because the specific activity (31.1 U mg^–1^) of rGluS toward CMC was approximately 3.7-, 2.8-, and 2.4-fold higher than that (8.3 U mg^–1^) of rGluL (Kim et al., 2022), that (11.2 U mg^–1^) of BpEG (Chen et al., 2020), and that (12.7 U mg^–1^) of *Xac*Cel8 (de Melo et al., 2021), respectively. In addition, the endo-β-1,4-glucanase activity (28.9 U mg^–1^) of rGluS for barley β-1,3-1,4-glucan was approximately 1.9- and 1.7-fold greater than that (15.1 U mg^–1^) of rGluL (Kim et al., 2022) and that (16.6 U mg^–1^) of *Xac*Cel8 (de Melo et al., 2021), respectively. However, while cold-adapted rGluS showed approximately 7.6-fold greater activity on CMC than thermostable GH8ErCel (Ontañon et al., 2019), its biocatalytic ability to degrade barley β-1,3-1,4-glucan was 42% lower compared to that of GH8ErCel for the same substrate. Furthermore, the specific activities of cold-adapted rGluS toward CMC and barley β-1,3-1,4-glucan, which were measured as 31.1 and 28.9 U mg^–1^, respectively, were lower than the corresponding values for mesophilic, multi-functional GH8 Cen219 (Zhang et al., 2013), which were estimated at 107.2 and 189.6 U mg^–1^.

The kinetic parameters (*V*_*max*_, *K*_*m*_, and *k*_*cat*_/*K*_*m*_) of cold-adapted rGluS with respect to CMC and barley β-1,3-1,4-glucan, which were determined via non-linear regression analysis using the Michaelis-Menten equation within a concentration range of 0.2%–1.2%, are presented in Table 3. Under optimal reaction conditions, rGluS showed a *V*_*max*_ value of 58.72 U mg^–1^ and a *K*_*m*_ value of 3.46 mg mL^1^ for CMC. However, the *V*_*max*_ and *K*_*m*_ values of rGluS for barley β-1,3-1,4-glucan were measured to be 53.89 U mg^–1^ and 3.89 mg mL^1^, respectively. It is also noteworthy that owing to its stronger substrate affinity and higher turnover number (*k*_*cat*_) for CMC, rGluS demonstrated a biocatalytic efficiency (*k*_*cat*_/*K*_*m*_: 11.02 mg^–1^ s^–1^ mL) approximately 1.25-fold higher than its *k*_*cat*_/*K*_*m*_ value (8.79 mg^–1^ s^–1^ mL) determined for barley β-1,3-1,4-glucan. These results suggested that compared to three previously characterized cold adapted GH8 endo-β-1,4-glucanases, rGluS was a notably effective cold-adapted GH8 homolog with superior biocatalytic efficiency against cellulosic substrates. Specifically, the *k*_*cat*_/*K*_*m*_ value (11.02 mg^–1^ s^–1^ mL) of rGluS toward CMC was evaluated to be approximately 4.40-, 3.89-, and 1.82-fold higher than that (2.50 mg^–1^ s^–1^ mL) of CEL8M (Bhat et al., 2013), that (2.83 mg^–1^ s^–1^ mL) of rGluL (Kim et al., 2022), and that (6.05 mg^–1^ s^–1^ mL) of *Xac*Cel8 (de Melo et al., 2021), respectively, toward the same substrate. Additionally, the *k*_*cat*_/*K*_*m*_ value (8.79 mg^–1^ s^–1^ mL) of rGluS for barley β-1,3-1,4-glucan was approximately 1.38- and 2.20-fold greater than that (6.35 mg^–1^ s^–1^ mL) of rGluL (Kim et al., 2022) and that (3.98 mg^–1^ s^–1^ mL) of *Xac*Cel8 (de Melo et al., 2021), respectively, when assayed against the identical polysaccharide.

**TABLE 3:** Kinetic parameters of rGluS for CMC and barley β-1,3-1,4-glucan.

Substrate	*V*_max_ (U mg^–1^)	*K*_m_ (mg mL^–1^)	*k*_cat_ (s^–1^)	*k*_cat_/*K*_m_ (mg^–1^ s^–1^ mL)
CMC	58.72	3.46	38.16	11.02
Barley β-1,3-1,4-glucan	53.89	3.98	35.02	8.79

The results of UHPLC analysis revealed that rGluS was unable to cleave either C_3_ or C_2_, implying the absence of both β-glucosidase and cellobiohydrolase activities (Table 4). The lack of rGluS activity on the two D-cellooligosaccharides was further supported the failure to observe cleavage of PNP-glucopyranoside and PNP-cellobioside (Table 2). These findings were consistent with results reported for several GH8 endo-β-1,4-glucanases, including *Xac*Cel8 (de Melo et al., 2021), GH8ErCel (Ontañon et al., 2019), celA1805 (Huang et al., 2021), Cel9Pa (Ghio et al., 2020), and BpEG (Chen et al., 2020), all of which lack hydrolytic activity even toward C_3_. Conversely, unlike rGluS, rGluL has been elucidated to have biocatalytic activity for cleaving C_3_, although it is unable to hydrolyze C_2_ (Kim et al., 2022). In this study, despite its inability to act on C_2_ and C_3_, rGluS was found to display strong hydrolytic activity toward C_5_, C_6_, and CMC; C_4_ was largely resistant, with evidence suggesting very slow cleavage by the enzyme (Table 4). Specifically, rGluS-mediated biocatalytic degradation of C_4_ caused the formation of a small amount of C_2_ as the sole end product. However, because the rate of hydrolysis was low due to its weak binding affinity to C_4_, a substantial proportion of the added C_4_ (94.2%) remained uncleaved. It is considered that compared to C_5_, C_6_, and CMC, the significantly lower hydrolysis rate of C_4_ might be attributed to the weak binding affinity of rGluS to the substrate. In this case, the C_4_-hydrolyzing capacity of rGluS was very comparable to the inability of *Xac*Cel8 (de Melo et al., 2021) and Egl-257 (Hakadama et al., 2002) to hydrolyze the same substrate. In addition, compared to rGluS, rGluL was found to catalyze the breakdown of C_4_ that generated a product mixture of C_1_ (3.8%), C_2_ (62.8%), C_3_ (31.1%), and C_4_ (2.3%) (Kim et al., 2022). It seemed that rGluS was highly active on C_5_ and C_6_ because all the given substrates were degraded by the enzyme to smaller molecules (C_1_, C_2_, and C_3_) during hydrolytic reactions (Table 4). Specifically, C_5_ was efficiently converted into a mixture comprising C_1_ (13.1%), C_3_ (14.6%), and C_2_ (72.3%) as the predominant product, with no formation of longer D-cellooligosaccharides with a degree of polymerization ≥ 6. Likewise, the end products of C_6_ hydrolysis by rGluS were identified to be C_2_ (42.6%) and C_3_ (48.0%) together with C_1_ (9.4%) as the minor product. A previous report has shown that in contrast to rGluS, rGluL-mediated hydrolysis of C_5_ yields a mixture solely of C_2_ (58.2%) and C_3_ (41.8%) without C_1_, although C_2_ is the principal product (Kim et al., 2022). Moreover, the prominent end products released from the degradation of C_6_ or CMC by rGluL (Kim et al., 2022) and rGluS were analyzed to be C_2_ and C_3_, respectively, reflecting their varying action on cellulosic materials. It is important to note that in contrast to rGluS, *Xac*Cel8 (de Melo et al., 2021), BpEG (Chen et al., 2020), and GH8ErCel (Ontañon et al., 2019) primarily released C_3_ as the end product from C_5_ hydrolysis. As displayed in Table 4, the analysis demonstrated that compared to C_5_ or C_6_, rGluS catalyzed the depolymerization of CMC to yield C_1_ (6.5%), C_2_ (18.7%), and C_4_ (2.8%) in addition to C_3_ (72.0%) identified as the principal hydrolysis product under the specified reaction conditions. This result revealed that the action mode of rGluS on CMC closely mirrored that of BpEG releasing exclusively C_1_ (Chen et al., 2020) and that of Cel8H releasing C_3_ and C_4_ (Huang et al., 2010), respectively, from the polymer. Furthermore, GH8ErCel (Ontañon et al., 2019), CEL8M (Bhat et al., 2013), and *Xac*Cel8 (de Melo et al., 2021) were shown to deconstruct CMC into a mixture of D-cellooligosaccharides with a degree of polymerization of ≥2, without generating C_1_ as the end product. Collectively, these findings elucidated by rGluS-catalyzed degradation reactions of D-cellooligosaccharides and CMC strongly supported the endo-acting nature of rGluS in the absence of transglycosylation activity.

**TABLE 4:** Liquid chromatography analysis of the degradation products of D-cellooligosaccharides and CMC by rGluS.

Substrate	Composition (%)^a^ of products formed by biocatalytic degradation			
	C_1_	C_2_	C_3_	C_4_
C_2_		100.0		
C_3_			100.0	
C_4_		5.8		94.2
C_5_	13.1	72.3	14.6	
C_6_	9.4	42.6	48.0	
CMC	6.5	18.7	72.0	2.8

^a^Liquid chromatography-mass spectrometry peak area percentage.

## 4 Conclusion

The non-modular GH8 endo-β-1,4-glucanase (GluS) from *Glacieibacterium* sp. PAMC 29367, an Antarctic psychrophilic bacterium associated with lichen symbiosis, which was discovered via an *in silico* analysis of its complete genome sequence, was genetically and biocatalytically characterized. Relative to previously described GH8 functional analogues (Table 1), rGluS represents a novel, low-temperature-active cellulosic biomass-disintegrating enzyme, and displays distinct features in its amino acid sequence, pH stability, thermal behavior of endo-β-1,4-glucanase activity, kinetic efficiency, and substrate-specific activity profile. The highly active, cold-adapted rGluS, demonstrating broad pH stability, holds significant promise as an efficient biocatalyst for low-temperature applications in food and textile processing. The findings of the present study provide insights into the ecological importance of fibrolytic psychrophiles and their cold-adapted cellulolytic enzymes involved in the biorecycling of cellulosic wastes in the frigid Antarctic environment.

## Data Availability

The datasets presented in this study can be found in online repositories. The names of the repository/repositories and accession number(s) can be found in the article/Supplementary material.

## Footnotes

1
https://www.cazy.org/Glycoside-Hydrolases.html

2
https://web.expasy.org/compute_pi/

3
https://services.healthtech.dtu.dk/services/SignalP-6.0/

4
https://www.cazy.org/

5
https://www.cazy.org/GH8_characterized.html

6
https://www.expasy.org/resources/compute-pi-mw

## References

[B1] Akbarian A. Andooz A. Kowsari E. Ramakrishna S. Asgari S. Cheshmeh Z. A. (2022). Challenges and opportunities of lignocellulosic biomass gasification in the path of circular bioeconomy. *Bioresour. Technol.* 362:127774. 10.1016/j.biortech.2022.127774 35964915

[B2] Al-Ghanayem A. A. Joseph B. (2020). Current perspective in using cold-active enzymes as eco-friendly detergent additive. *Appl. Microbiol. Biotechnol.* 104 2871–2882. 10.1007/s00253-020-10429-x 32037467

[B3] Arneen F. Moslem M. A. Hadi S. Al-Sabri A. E. (2014). Biodegradation of cellulosic materials by marine fungi isolated from south corniche of Jeddah, Saudi Arabia. *J. Pure Appl. Microbiol.* 8 3617–3626.

[B4] Bates S. T. Cropsey G. W. G. Caporaso J. G. Knight R. Fierer N. (2011). Bacterial communities associated with the lichen symbiosis. *Appl. Environ. Microbiol.* 77 1309–1314. 10.1128/AEM.02257-10 21169444 PMC3067232

[B5] Bélguin P. Cornet P. Aubert J. P. (1985). Sequence of a cellulase gene of the thermophilic bacterium *Clostridium thermocellum*. *J. Bacteriol.* 162 102–105. 10.1128/jb.162.1.102-105.1985 3980433 PMC218960

[B6] Bhardwaj N. Kumar B. Agrawal K. Verma O. (2021). Current perspective on production and applications of microbial cellulases: A review. *Bioresour. Bioprocess.* 8:95. 10.1186/s40643-021-00447-6 38650192 PMC10992179

[B7] Bhat A. Riyaz-Ul-Hassan S. Ahmad N. Srivastava N. Johri S. (2013). Isolation of cold-active, acidic endocellulase from Ladakh soil by functional metagenomics. *Extremophiles* 17 229–239. 10.1007/s00792-012-0510-8 23354361

[B8] Cai J. He Y. Yu X. Banks S. W. Yang Y. Zhang X. (2017). Review of physicochemical properties and analytical characterization of lignocellulosic biomass. *Renew. Sustain. Energy Rev.* 76 309–322. 10.1016/j.rser.2017.03.072

[B9] Cano-Ramírez C. Santiago-Hernández A. Rivera-Orduña F. N. García-Huante Y. Zúñiga G. Hidalgo-Lara M. E. (2016). Expression, purification and characterization of an endoglucanase from *Serratia proteamaculans* CDBB-1961, isolated from the gut of *Dendroctonus adjunctus* (Coleoptera: Scolytinae). *AMB Expr.* 6:63. 10.1186/s13568-016-0233-9 27576896 PMC5005244

[B10] Chen F. Ye J. Kameshwar A. K. S. Wu X. Ren J. Qin W. (2020). A novel cold-adaptive endo-1,4-β-glucanase from *Burkholderia pyrrocinia* JK-SH007: Gene expression and characterization of the enzyme and mode of action. *Front. Microbiol.* 10:3137. 10.3389/fmicb.2019.03137 32038571 PMC6987409

[B11] de Melo R. R. de Lima E. A. Persinoti G. F. Vieira P. S. de Sousa A. S. Zanphorlin L. M. (2021). Identification of a cold-adapted and metal-stimulated β-1,4-glucanase with potential use in the extraction of bioactive compounds from plants. *Int. J. Biol. Macromol.* 166 190–199. 10.1016/j.ijbiomac.2020.10.137 33164774

[B12] de Menezes A. B. Lockhart R. J. Cox M. J. Allison H. E. McCarthy A. J. (2008). Cellulose degradation by micromonosporas recovered from freshwater lakes and classification of these actinomycetes by DNA gyrase B gene sequencing. *Appl. Environ. Microbiol.* 74 7080–7084. 10.1128/AEM.01092-08 18820070 PMC2583502

[B13] Dimarogona M. Topakas E. Christakopoulos P. (2013). Recalcitrant polysaccharide degradation by novel oxidative biocatalysts. *Appl. Microbiol. Biotechnol.* 97 8455–8465. 10.1007/s00253-013-5197-y 23995228

[B14] Dong M. Yang Y. Tang X. Shen J. Xu B. Li J. (2016). NaCl-, protease-tolerant and cold-active endoglucanase from *Paenibacillus* sp. YD236 isolated from the feces of *Bos frontalis*. *SpringerPlus* 5:746. 10.1186/s40064-016-2360-9 27376014 PMC4909688

[B15] Duncan S. M. Minasaki R. Farrell R. L. Thwaites J. M. Held B. W. Arenz B. E. (2008). Screening fungi isolated from historic *Discovery* Hut on Ross Island, Antarctica for cellulose degradation. *Antarct. Sci.* 20 463–470. 10.1017/S0954102008001314

[B16] Ghio S. Bradanini M. B. Garrido M. M. Ontañon O. M. Piccinni F. E. de Villegas R. M. D. (2020). Synergistic activity of Cel8Pa β-1,4 endoglucanase and Bg1Pa β-glucosidase from *Paenibacillus xylanivorans* A59 in beta-glucan conversion. *Biotechnol. Rep.* 28:e00526. 10.1016/j.btre.2020.e00526 32963976 PMC7490527

[B17] Hakadama Y. Endo K. Takizawa S. Kobayashi T. Shirai T. Yamane T. (2002). Enzymatic properties, crystallization, and deduced amino acid sequence of an alkaline endoglucanase from *Bacillus circulans*. *Biochim. Biophys. Acta* 1570 174–180. 10.1016/s0304-4165(02)00194-0 12020807

[B18] Hall M. Bansal P. Lee J. H. Realff M. J. Bommarius A. S. (2010). Cellulose crystallinity – a key predictor of the enzymatic hydrolysis rate. *FEBS J.* 277 1571–1582. 10.1111/j.1742-4658.2010.07585.x 20148968

[B19] Handique G. Phukan A. Bhattacharyya B. Baruah A. A. L. H. Rahnan S. W. Baruah R. (2017). Characterization of cellulose degrading bacteria from the larval gut of the white grub beetle *Lepidiota mansueta* (Coleoptera: Scarabaeidae). *Arch. Insect Biochem. Physiol.* 94:e21370. 10.1002/arch.21370 28094878

[B20] Horn S. J. Vaaje-Kolstad G. Westereng B. Eijsink V. G. H. (2012). Novel enzymes for the degradation of cellulose. *Biotechnol. Biofuels* 5:45. 10.1186/1754-6834-5-45 22747961 PMC3492096

[B21] Huang X. Shao Z. Hong Y. Lin L. Li C. Huang F. (2010). Cel8H, a novel endoglucanase from the halophilic bacterium *Halomonas* sp. S66-4: Molecular cloning, heterogonous expression, and biochemical characterization. *J. Microbiol.* 48 318–324. 10.1007/s12275-009-0188-5 20571949

[B22] Huang Z. Ni G. Zhao X. Wang F. Qu M. (2021). Characterization of a GH8 β-1,4-glucanase from *Bacillus subtilis* B111 and its saccharification potential for agricultural straws. *J. Microbiol. Biotechnol.* 31 1446–1454. 10.4014/jmb.2105.05026 34409950 PMC9705894

[B23] Jin X. Wang J.-K. Wang Q. (2023). Microbial β-glucanases: Production, properties, and engineering. *World J. Microbiol. Biotechnol.* 39:106. 10.1007/s11274-023-03550-2 36847914

[B24] Kim D. Y. Kim J. Lee Y. M. Byeon S. M. Gwak J. H. Lee J. S. (2022). Novel. acidic, and cold-adapted glycoside hydrolase family 8 endo-β-1,4-glucanase from an Antarctic lichen-associated bacterium, *Lichenicola cladoniae* PAMC 26568. *Front. Microbiol.* 13:935497. 10.3389/fmicb.2022.935497 35910630 PMC9329076

[B25] Kim D. Y. Lee M. J. Cho H.-Y. Lee J. S. Lee M.-H. Chung C. W. (2016). Genetic and functional characterization of an extracellular modular GH6 endo-β-1,4-glucanase from an earthworm symbiont, *Cellulosimicrobium funkei* HY-13. *Antonie van Leeuwenhoek* 109 1–12. 10.1007/s10482-015-0604-2 26481128

[B26] Liu L. Huang W.-C. Liu Y. Li M. (2021). Diversity of cellulolytic microorganisms and microbial cellulases. *Int. Biodeterior. Biodegrad.* 163:105277. 10.1016/j.ibiod.2021.105277

[B27] López-Mondéjar R. Zühlke D. Becher D. Riedel K. Baldrian P. (2016). Cellulose and hemicellulose decomposition by forest soil bacteria proceeds by the action of structurally variable enzymatic systems. *Sci. Rep.* 6:25279. 10.1038/srep25279 27125755 PMC4850484

[B28] Lv W. Yu Z. (2013). Isolation and characterization of two thermophilic cellulolytic strains of *Clostridium thermocellum* from a compost sample. *J. Appl. Microbiol.* 114 1001–1007. 10.1111/jam.12112 23279216

[B29] Miroshnichenko M. L. Kublanov I. V. Kostrikina N. A. Tourova T. P. Kolganova T. V. Birkeland N. K. (2008). *Caldicellulosiruptor kronotskyensis* sp. nov. and *Caldicellulosiruptor hydrothermalis* sp. nov., two extremely thermophilic, cellulolytic, anaerobic bacteria from Kamchatka thermal springs. *Int. J. Syst. Evol. Microbiol.* 58 1492–1496. 10.1099/ijs.0.65236-0 18523201

[B30] Moraïs S. David Y. B. Bensoussan L. Duncan S. H. Koropatkin N. M. Martens E. C. (2016). Enzymatic profiling of cellulosomal enzymes from the human gut bacterium, *Ruminococcus champanellensis*, reveals a fine-tuned system for cohesion-dockerin recognition. *Environ. Microbiol.* 18 542–556. 10.1111/1462-2920.13047 26347002

[B31] Noh H.-J. Park Y. Hong S. G. Lee Y. M. (2021). Diversity and physiological characteristics of Antarctic lichens-associated bacteria. *Microorganisms* 9:607. 10.3390/microorganisms9030607 33804278 PMC8001610

[B32] Ontañon O. M. Ghio S. de Villegas R. M. D. Garrido M. M. Talia P. M. Fehér C. (2019). A thermostable GH8 endoglucanase of *Enterobacter* sp. R1 is suitable for β-glucan deconstruction. *Food Chem.* 298:124999. 10.1016/j.foodchem.2019.124999 31261010

[B33] Parvizpour S. Razmara J. Jomah A. F. Shamsir M. S. Illias R. M. (2015). Structural prediction of a novel laminarinase from the psychrophilic *Glaciozyma antarctica* PI12 and its temperature adaptation analysis. *J. Mol. Model.* 21:63. 10.1007/s00894-015-2617-1 25721655

[B34] Santiago M. Ramirez-Sarmiento C. A. Zamora R. A. Parra L. P. (2016). Discovery, molecular mechanisms, and industrial applications of cold-active enzymes. *Front. Microbiol.* 7:1408. 10.3389/fmicb.2016.01408 27667987 PMC5016527

[B35] Singh A. Upadhyay V. Upadhyay A. K. Singh S. M. Panda A. K. (2015). Protein recovery from inclusion bodies of *Escherichia coli* using mild solubilization process. *Microb. Cell Fact.* 14:41. 10.1186/s12934-015-0222-8 25889252 PMC4379949

[B36] Song J. M. Hong S. K. An Y. J. Kang M. H. Hong K. H. Lee Y.-H. (2017). Genetic and structural characterization of a thermo-tolerant, cold-active, and acidic endo-β-1,4-glucanase from Antarctic springtail, *Cryptopygus antarcticus*. *J. Agric. Food Chem.* 65 1630–1640. 10.1021/acs.jafc.6b05037 28156112

[B37] Sutaoney P. Rai S. N. Shinha S. Choudhary R. Gupta A. K. Singh S. K. (2024). Current perspective in research and industrial applications of microbial cellulases. *Int. J. Biol. Macromol.* 264:130639. 10.1016/j.ijbiomac.2024.130639 38453122

[B38] Tamura K. Stecher G. Kumar S. (2021). MEGA11: Molecular evolutionary genetics analysis version 11. *Mol. Biol. Evol.* 38 3022–3027. 10.1093/molbev/msab120 33892491 PMC8233496

[B39] Ueda M. Ito A. Nakazawa M. Miyatake K. Sakaguchi M. Inouye K. (2014). Cloning and expression of the cold-adapted endo-1,4-β-glucanase gene from *Eisenia fetida*. *Carbohyd. Polym.* 101 511–516. 10.1016/j.carbpol.2013.09.057 24299806

[B40] van Solingen P. Meijer D. van der Kleij W. A. H. Barnett C. Bolle R. Power S. D. (2001). Cloning and expression of an endocellulase gene from a novel streptomycete isolated from an East African soda lake. *Extremophiles* 5 333–341. 10.1007/s007920100198 11699647

[B41] Vassilev S. V. Baxter D. Anderson L. K. Vassileva C. G. Morgan T. J. (2012). An overview of the organic and inorganic phase composition of biomass. *Fuel* 94 1–33. 10.1016/j.fuel.2011.09.030

[B42] Vester J. K. Glaring M. A. Stougaard P. (2014). Discovery of novel enzymes with industrial potential from a cold and alkaline environment by a combination of functional metagenomics and culturing. *Microb. Cell Fact.* 13 2–14. 10.1186/1475-2859-13-72 24886068 PMC4035831

[B43] Wierzbicka-Woś A. Henneberger R. Batista-García R. A. Martínez-Ávila L. Jackson S. A. Kennedy J. (2019). Biochemical characterization of a novel monospecific endo-β-1,4-glucanase belonging to GH family 5 from a rhizosphere metagenomics library. *Front. Microbiol.* 10:1342. 10.3389/fmicb.2019.01342 31258522 PMC6587912

[B44] Yang J. Dang H. (2011). Cloning and characterization of a novel cold-active endoglucanase establishing a new subfamily of glycosyl hydrolase family 5 from a psychrophilic deep-sea bacterium. *FEMS Microbiol. Lett.* 325 71–76. 10.1111/j.1574-6968.2011.02413.x 22092864

[B45] Zeng R. Xiong P. J. Wen J. (2006). Characterization and gene cloning of a cold-active cellulase from a deep-sea psychrotrophic bacterium *Pseudoalteromonas* sp. DY3. *Extremophiles* 10 79–82. 10.1007/s00792-005-0475-y 16133657

[B46] Zhang L. Fan Y. Zheng H. Du F. Zhang K.-Q. Huang X. (2013). Isolation and characterization of a novel endoglucanase from a *Bursaphelenchus xylophilus* metagenomics library. *PLoS One* 8:e82437. 10.1371/journal.pone.0082437 24386096 PMC3873927

[B47] Zhao F. Cao H.-Y. Zhao L.-S. Zhang Y. Li C.-Y. Zhang Y.-Z. (2019). A novel subfamily of endo-β-1,4-glucanases in glycoside hydrolase family 10. *Appl. Environ. Microbiol.* 85:e01029-19. 10.1128/AEM.01029-19 31253686 PMC6715846

[B48] Zolotnitsky G. Cogan U. Adir N. Solomon V. Shoham G. Shoham Y. (2004). Mapping glycoside hydrolase substrate subsites by isothermal titration calorimetry. *Proc. Natl. Acad. Sci. U S A.* 101 11275–11280. 10.1073/pnas.0404311101 15277671 PMC509194

