# Supplementary material for: Identification and characterization of a novel, low-temperature-active GH8 endo-β-1,4-glucanase exhibiting broad pH stability from Antarctic Glacieibacterium sp. PAMC 29367
Source: Front Microbiol. 2025 Oct 21;16:1682092. doi: 10.3389/fmicb.2025.1682092 (PMC12583906; doi:10.3389/fmicb.2025.1682092)
Supplement: Supplementary file 1 [file Data_Sheet_1.pdf]

GH family 5

>Acidothermus cellulolyticus (AAA75477)

LDANNVPVRIAGINWFGFETCNYVVHGLWSRDYRSMLDQIKSLGYNTIRLPYSDDILKPGTMPNSINFYQMNQDL
QGLTSLQVMDKIVAYAGQIGLRIILDRHRPDCSGQSALWYTSSVSEATWISDLQALAQRYKGNPTVVGFDLHNEP
HDPACWGCGDPSIDWRLAAERAGNAVLSVNPNLLIFVEGVQSYNGDSYWWGGNLQGAGQYPVVLNVPNRLVYSAH
DYATSVYPQTWFSDPTFPNNMPGIWNKNWGYLFNQNIAPVWLGEFGTTLQSTTDQTWLKTLVQYLRPTAQYGADS
FQWTFWSWNPDS

>Cellulomonas fimi (AAA23089)

VDSTGKEAILSGVNWFGFNASERVFHGLWSGNITQITQQMAQRGINVVRVPVSTQLLLEWKAGTFLKPNVNTYAN
PELEGKNSLQIFEYWLTLCQKYGIKVFLDVHSAEADNSGHVYNMWWKGDITTEDVYEGWEWAATRWKDDDTIVGA
DIKNEPHGTQGSTERAKWDGTTDKDNFKHFAETASKKILAINPNWLVFVEGVEIYPKPGVPWTSTGLTDYYGTWW
GGNLRGVRDHPIDLGAHQDQLVYSPHDYGPLVFDQKWFQKDFDKASLTADVWGPNWLFIHDEDIAPLLIGEWGGR
LGQDPRQDKWMAALRDLVAERRLSQTFWVLNPNS

>Bacillus licheniformis DSM 13 (AAU23613)

VNQNGKAVQLKGISSHGLQWYGDYVNKDSLKWLRDDWGINVFRAAMYTAEGGYIDNPSVKNKVKEAVEAAKELGI
YVIIDWHILSDGNPNQNKAKAKEFFNEMSRLYGKTPNVIFEIANEPNGDVNWNRDIKPYAEEILSVIRKNSPKNI
VIVGTGTWSQDVNDAADNQLKDGNVMYALHFYAGTHGQSLRDKADYALSKGAPIFVTEWGTSDASGNGGVYLDQS
REWLKYLDSKKISWVNWNLSDK

>Caldicellulosiruptor bescii DSM 6725 (CAB01405)

DQKGEIIQLRGMSTHGLQWYGDIINKNAFKALSKDWECNVIRLAMYVGEGGYASNPSIKEKVIEGIKLAIENDMY
VIVDWHVLNPGDPNAEIYKGAKDFFKEIATSFPNDYHIIYELCNEPNPNEPGVENSLDGWKKVKAYAQPIIKMLR
SLGNQNIIIVGSPNWSQRPDFAIQDPINDKNVMYSVHFYSGTHKVDGYVFENMKNAFENGVPIFVSEWGTSLASG
DGGPYLDEADKWLEYLNSNYISWVNWSLSNKN

>Geobacillus sp. 70PC53 (ACJ60856)

LNQHNKPFQLRGISTHGLQWFGQFANKDAFQTLRDDWKANVVRLAMYTDPNANGYIAQPEWLKAKVKEGVQAALD
LGMYVIIDWHILNDNDPNLYKEQAKRFFAEMAREYGKYPNVIYEIANEPNGNDVTWEEKIRPYADEVIRTIRSID
RDNLIIVGTGTWSQDVDDVASDPLPYKNIMYAVHFYSGTHTQWLRDRVDAALQAGTPVFVSEWGTSDASGDGGPY
LEEAEKWIEFLNERGISWVNWSLCDKN

GH family 6

>Cellulosimicrobium funkei (ALK04194)

MRRRAVLSAVAALALASPLLAAAGSAGAAPVPTPAVPRATAAPAADDVVGPGTRLYVDPFSTTLQAAAVLTGQAR
ADAQLLGSFPSASWFTGGTPDEVRDDVASVVRAAATDGSVPTVVVYNLPFRDCAQYSAGGAADTAAYTAWVDAVA
EGIGDDPAIVVLEPDGLGIIPWYTDINGNPEWCRPAELDPETAAADRFVQLNHAVDALTALPATSVYLDGTHSGW
LGVGDITDRLIKAGVERADGVFVNASNYVETERLVKYGTWISDCVNLSVNSWWEPAWCASQYYPANPDDVETWGL
TDAAYAQAYADTGVVRDPAAQKHVVVDTSRNGQGPWTAPSGVYADPEVWCNPPDRGLGERPTTDTADPFVDAYLW
IKVPGESDGKCYRGTGGPLDPARGIEDPAAGQWFVEQAAELLALASPAVERPTCEVAYTVHGTWQGKKGNGFIAQ

VELRNTGASPLDGWELRWTPDGDQRLTDVWGAQAGRDGAALTAHSLSWNARVRPGATATFGFVASGAPGAGPQLV
TLGGRPCHVV

>Streptomyces halstedii (CAA78145)

MSRKLRTLMAALCALPLAFAAAPPAHAADPTTMTNGFYADPDSSASRWAAANPGDGRAAAINASIANTPMARWFG
SWSGAIGTAAGAYAGAADGRDKLPILVAYNIYNRDYCGGHSAGGAASPSAYADWIARFAGGIAARPAVVILEPDS
LGDYGCMNPAQIDEREAMLTNALVQFNRQAPNTWVYMDAGNPRWADAATMARRLHEAGLRQAHGFSLNVSNYITT
AENTAYGNAVNNELAARYGYTKPFVVDTSRNGNGSNGEWCNPSGRRIGTPTRTGGGAEMLLWIKTPGESDGNCGV
GSGSTAGQFLPEVAYKMIYGY

>Thermobifida fusca YX (AAC06388)

MSPRPLRALLGAAAAALVSAAALAFPSQAAANDSPFYVNPNMSSAEWVRNNPNDPRTPVIRDRIASVPQGTWFAH
HNPGQITGQVDALMSAAQAAGKIPILVVYNAPGRDCGNHSSGGAPSHSAYRSWIDEFAAGLKNRPAYIIVEPDLI
SLMSSCMQHVQQEVLETMAYAGKALKAGSSQARIYFDAGHSAWHSPAQMASWLQQADISNSAHGIATNTSNYRWT
ADEVAYAKAVLSAIGNPSLRAVIDTSRNGNGPAGNEWCDPSGRAIGTPSTTNTGDPMIDAFLWIKLPGEADGCIA
GAGQFVPQAAYEMAIAAGGTNPNPNPNPTPTPTPTPTPPPGSSGACTATYTIANEWNDGFQATVTVTANQNITGW
TVTWTFTDGQTITNAWNADVSTSGSSVTARNVGHNGTLSQGASTEFGFVGSKGNSNSVPTLTCAAS

>Xylanimicrobium pachnodae (AAD54679)

MHPPTRIRARAGVAAAAALAAALVATPASAHPGKSADGGEELWVNPKSSITEHLRTEHLKGQDRADAEALAGYAS
ASWFTGGTPSEVRKDVRDQVRRADRADAVPTLVAYNLPYRDCAQYSAGGAASQADYEEWIDAFAAGIGNERAIVI
LEPDGLGVIPWYTTIDGAQEWCQPEDADPATAAAERFAMFNHAVDAFGALPNAQVYLDAGNSAWLNVGENTDRLI
KAGVQRADGFFLNASNYQFTENSTAYGHWISSCIEVITRGLGAAADCGNQYWNGGPANDWTGVAMTRYAPWTAGN
ADPAADTSGVDSKYAQQLGDIVPTTQFVIDTSRNGVGPWDPTTSDVEYTGDAEDWCNPPDRGLGARPTLDVDDPL
VAGYLWIKVPGESDGQCYRSLGGPLDPERGMQDPAAGQWFAEQARELIELAVPPLETTRADCRGKGKGNGSADAN
GKGQANGKGNAGGNGHGQGHGGV

>Neisseria sicca (BAJ21449)

MSARRTASALLTAAALSVTGLTAVREHRDAADGCRVDYTVQSQWNSGFQGDVKITNLGSPLSSWTVAWTFPSSGQ
RVSQSWNANVTTSGSRVSASSIGWNGSLGTGASTQFGFIGSFTDSNPVPTSFTVNGVACTGTVTPTQGPTQGPTQ
GPTQGPTQGPTQGPTQGPTQGPTQNPQPSGDLYVDTENQSYAAWQAASGSTKDLLAKIALTPQSVWIGNWNSPSV
SQQQMRDATSRASAAGKTVSVVIYAIPGRDCGSYSGGRVAESEYAQWIDTVASGIVGKPIVILEPDALAQLGNCG
GQGDRVGYLRYAAKSLTAAGGVYIDAGHSGWLSVDTAVNRLNQIGFQYALAFALNTSNYQTTSASQQYGEQISQR
LGGKGYVIDTSRNGNGSNGEWCNRGRALGDKPRLVNDSTGLDALLWVKLPGESDGTCKRPAAGQWWQDMALEYAR
NAKW

GH family 7

>Aspergillus nidulans FGSC A4 (EAA63386)

MALLLSLSLLATTISAQQIGTPEIRPRLTTYHCTSANGCTEQNTSVVLDAATHPIHDASNPSVSCTTSNGLNPAL
CPDKQTCADNCVIDGITDYAAHGVETHGSRLTLTQYRNVNGALSSVSPRVYLVDESDPDEQEYRALSLLAQEFTF
TVNVSALPCGMNGALYLSEMSPSGGRSALNPAGASYGTGYCDAQCYVNPWINGEGNINGYGACCNEMDIWEANSR
STGFTPHACLYEPEETEGRGVYECASEDECDSAGENDGICDKWGCGFNPYALGNTEYYGRGQGFEVDTKEPFTVV
TQFLTDDGTSTGALTEIRRLYIQNGQVIENAVVSSGADSLTDSLCASTASWFDSYGGMEGMGRALGRGMVLAMSI
WNDAGGYMQWLDGGDAGPCNATEGAPEFIEEHTPWTRVVFEDLKWGDIGSTFQAS

>Trichoderma reesei (AAA34212)

MAPSVTLPLTTAILAIARLVAAQQPGTSTPEVHPKLTTYKCTKSGGCVAQDTSVVLDWNYRWMHDANYNSCTVNG
GVNTTLCPDEATCGKNCFIEGVDYAASGVTTSGSSLTMNQYMPSSSGGYSSVSPRLYLLDSDGEYVMLKLNGQEL
SFDVDLSALPCGENGSLYLSQMDENGGANQYNTAGANYGSGYCDAQCPVQTWRNGTLNTSHQGFCCNEMDILEGN
SRANALTPHSCTATACDSAGCGFNPYGSGYKSYYGPGDTVDTSKTFTIITQFNTDNGSPSGNLVSITRKYQQNGV
DIPSAQPGGDTISSCPSASAYGGLATMGKALSSGMVLVFSIWNDNSQYMNWLDSGNAGPCSSTEGNPSNILANNP
NTHVVFSNIRWGDIGSTTNSTAPPPPPASSTTFSTTRRSSTTSSSPSCTQTHWGQCGGIGYSGCKTCTSGTTCQY
SNDYYSQCL

>Trichoderma longibrachiatum (CAA43059)

MAPSATLPLTTAILAIGRLVAAQQPGTSTPEVHPKLTTYKCTTSGGCVAQDTSVVLDWNYRWMHDANYNSCTVNG
GVNTTLCPDEATCGKNCYIEGVDYAASGVTASGSTLTLNQYMPSSSGGYSSVSPRLYLLGPDGEYVMLKLNGQEL
SFDVDLSALPCGENGSLYLSQMDENGGANQYNTAGANYGSGYCDAQCPVQTWRNGTLNTSGQGFCCNEMDILEGN
SRANALTPHSCTATACDSAGCGFNPYGSGYPNYFGPGDTVDTSKTFTIITQFNTDNGSPSGNLVSITRKYRQNGV
DIPSAKPGGDTISSCPSASAYGGLATMGKALSSGMVLVFSIWNDNSQYMNWLDSGRAGPCSSTEGNPSNILANNP
GTHVVYSNIRWGDIGSTTNSTGGNPPPPPPPASSTTFSTTRRSSTTSSSPSCTQTHWGQCGGIGYTGCKTCTSGT
TCQYGNDYYSQCL

GH family 8

>Glacieibacterium sp. PAMC 29367 (PV751198)

PEDWKRFKAAFLHSDGRIVDTGNGGISHSEGQGYGMLLAETAADQEAFNSLFGWTEQVLARKDVALYSWRYVPKD
AVPVADTNNATDGDILIAWALMRAYDRWHRPEYRDRAHQIRSAIRTRLVSAQDGRTLLLPALVGFTAPDRTTVNP
SYYIWPALDLFARADGADGGWSALIADGERLAGEARFGPSRLPTDWVDVTSGGVAPAAGKPPRFGFDAVRVPLYQ
LMGGRRALTSDVAAYWSGFAGRGARIPAWVDVVTGEVAEYGLSEGGLAVVHRLVGAAGTGPPPGASRDYYSDVLA
LLTQI

>Polymorphobacter multimanifer (WP_184199733)

SLSSASDWQAFSAVYLRPDGRIVDNGNGAISHSEGQGYGMLLAEAAADRAAFDSMWGWTEKTLARSDVALYSWRY
SPGEAVPVSDPNNATDGDILIAWALLRAHARWRQPEHMARAREIATTIRTKLIHRQAGRSLLLPALTGFVRPDRT
TINPCYYIWPAIDLFRRIDPSDAWEALARDGERLARDARFGPSRLPCDWVDVTATGVVLPAADKPPRFGFDAIRI
PLYQLAGNRRSLATEVVSYWRQLIERKTMIPAWIDVVSGERAPYALSPGGTAMVGHLLHASDMVPASLPVLGSDY
YSDV

>Sphingomonas colocasiae (MBY8824380)

GWDAFKARYLLSEGRIADSGNGGISHSEGQGYAMLLAEITGDRDSFDRLHGWTETTLIRPHEALFSWRFEPGKGV
TDPNNATDGDILIAWALMRAAVRWRDARYGARATAIRAAIHAHLVRKQGSRTVLLPGLAGFDHAGRTTINLSYYI
WPALDAFRKADGADRWAAVIADGEKLLVEARAGPLQLPTDWTDIGADGHAAPAADKPPRFGFDAIRIPLYLALGN
RRAGAETIARFWRSYAEPGKPIPAWVDVKTGEVAPYPLSDGGYALVRRLLGPAAPMRPEASPADYYSTVLKLLAQ
I

>Sphingomonadales bacterium 63-6 (OJW72739)

WQRYRAAFLSPDGRIIDTGNGGISHSEGQGYGMILAFAASDRDAFASIYHWTEQSLSRGDMALFSWRYDPREINP

VGDPNNATDGDMLIAWALALAGKRWKMQHYLDRSAQIRNAIRSRCVTSQYGRQLLLPGIQGFVTPAGVTVNPSYF
IWPALDTFAALDGQHVWGQLIADCEDLVRLAKFGAHRLPPDWLTVTGTSQVIPAASHPPRFGFDAIRIALYSLMG
MRANLAADIGTFWRQRLTQQRPIPAWIDVVTGEEAPYPVSEGGAAIVGRLLGAPEPATLSADYFAASLQML

>Xanthomonadaceae bacterium (RYD28981)

TGTNGVSWATFKQRFVADDGRIVDNGNGDVSHSEGQSYGLLLSQAAGDRVAFDKILGWTEAKLARPDVPLYVWRY
DPRSANPVADRNNATDGDLLIAYALAEATKRWHEPRYAARSRVIVDAIATRLVKTVGGRMILLPGLDGFSTAART
TINPCYYVWPALDLFAKTGNAAVWRQVITDGEALIAASQFGAHRLPTDWIDVSNVGAVSPAVDKPPRFGFDAIRV
PLYANVSGRNALVVPIRTFWRQLPVERIPAWTNVITGQVAEYPLSTGGQAVVNRVLGTPSTHVLSTDYYAAALQC
L

>Lichenicola cladoniae PAMC 26568 (ON016586)

QWQSYRSRFIRPDGRVVDTGNRDVSHSEGQGYGLLFAEHFDDQATFDSILTWTRKTLQCRPDRLHAWRFDPGAQP
PVGDLNNATDGDLLIAMALARAARRWNRPELREQARSIYADVLRLATREVGGRLVLLPGVTGFVTPSGTEINLSY
LIFPSLIEASSLGDQARWRRVVHDGVGLIDIARFGRWDLPPDWLRLNPPHAPVPAPGKPPRFSYDAVRIPLYLEW
SGLLTAPLLNRFRTYWSAFGDGLPAWVDLATNARAPYPAPSGFHAVAECTGLVLPPGSMPAAFPSVASDPDYYSA
SLTLLARI

GH family 9

>Acetivibrio thermocellus ATCC 27405 (ABN51779)

MKKILAFLLTVALVAVVAIPQAVVSFAADFNYGEALQKAIMFYEFQRSGKLPENKRNNWRGDSALNDGADNGLDL
TGGWYDAGDHVKFNLPMAYAVTMLAWSVYESRDAYVQSGQLPYILDNIKWATDYFIKCHPSPNVYYYQVGDGALD
HSWWGPAEVMQMPRPSFKVDLTNPGSTVVAETAAAMAASSIVFKPTDPEYAATLLRHAKELFTFADTTRSDAGYR
AAEGYYSSHSGFYDELTWASIWLYLATGDQSYLDKAESYEPHWERERGTTLISYSWAHCWDNKLYGSLLLLAKIT
GKSYYKQCIENHLDYWTVGFNGSRVQYTPKGLAYLDRWGSLRYATTQAFLASVYADWSGCDPAKAAVYKEFAKKQ
VDYALGSTGRSFVVGFGKNPPRNPHHRTAHSSWSALMTEPAECRHILVGALVGGPDGSDSYVDRLDDYQCNEVAN
DYNAGFVGALAKMYEKYGGEPIPNFVAFETPGEEFYVEAAVNAAGPGFVNIKASIINKSGWPARGSDKLSAKYFV
DISEAVAKGITLDQITVQSTTNGGAKVSQLLPWDPDNHIYYVNIDFTGINIFPGGINEYKRDVYFTITAPYGEGN
WDNTNDFSFQGLEQGFTSKKTEYIPLYDGNVRVWGKVPDGGSEPDPTPTITVGPTPSVTPTSVPGIMLGDVNFDG
RINSTDYSRLKRYVIKSLEFTDPEEHQKFIAAADVDGNGRINSTDLYVLNRYILKLIEKFPAEQ

>Cellulomonas fimi (AAA23086)

MLRQVPRTLVAGGSALAVAVGVLVAPLATGAAAAPTYNYAEALQKSMFFYQAQRSGDLPADFPVSWRGDSGLTDG
ADVGKDLTGGWYDAGDHVKFGFPMAFSATMLAWGAIESPTGYSKAGSLDELKDNLRFVSDYFVKAHTAPNELYVQ
VGDGEADHKWWGPAEVMTMARPSHKISASCPGSDVAAETAAALASSAIVLKGDDPAYAATLVSHAKQLYTFADTY
RGAYSDCVTAASAYYKSWSGYQDELVWGAYWLYKATGDATYLAKAEAEYDKLGTENQSTTRSYKWTIAWDNKQFG
TYALLAMETGKQKYVDDANRWLDYWTVGVNGQKVPYSPGGQAVLDSWGALRYAANTSFVALVYSDWMTDATRKAR
YHDFGVRQINYALGDNPRSSSYVVGFGANPPTAPHHRTAHGSWLDSITTPAQSRHVLYGALVGGPGSPNDAYTDS
RQDYVANEVATDYNAGFTSALARLVEEYGGTPLASFPTPEQPDGDQLFVEAMLNQPPSGTFTEVKAMIRNQSAFP
ARSLKNAKVRYWFTTDGFAASDVTLSANYSECGAQSGKGVSAGGTLGYVELSCVGQDIHPGGQSQHRREIQFRLT
GPAGWNPANDPSYTGLTQTALAKASAITLYDGSTLVWGKEPTGTTTDTTPPTTPGTPVATGVTTVGASLSWAAST
DAGSGVAGYELYRVQGTTQTLVGTTTAAAYILRDLTPGTAYSYVVKAKDVAGNVSAASAAVTFTTDTTGETEPPT
TPGTPVASAVTSTGATLAWAPSTGDPAVSGYDVLRVQGTTTTVVAQTTVPTVTLSGLTPSTAYTYAVRAKNVAGD
VSALSAPVTFTTAAPPVDTVAPTVPGTPVASNVATTGATLTWTASTDSGGSGLAGYEVLRVSGTTQTLVASPTTA
TVALAGLTPATAYSYVVRAKDGAGNVSAVSSPVTFTTLPVTSTPSCTVVYSTNSWNVGFTGSVKITNTGTTPLTW
TLGFAFPSGQQVTQGWSATWSQTGTTVTATGLSWNATLQPGQSTDIGFNGSHPGTNTNPASFTVNGEVCG

>Cellvibrio japonicus Ueda107 (ACE85757)

MINRSVLKIPALVKPLVQALVLVGCTLGVAQAEVGNPRVNQLGYIPNGDRIAVYKASNNSAQTWQLTHNGSLIAS
GQTIPKGSDASSGDNIHHIDLSSVTATGSGFTLTVGGDSSYPFSISSTTFNAAFYDALKYFYHNRSGIAIETPYT
GGGRGSYASHSRWSRPAGHLNQGANKGDMNVPCWSGTCNYSLNVTKGWYDAGDHGKYVVNGGISVWTLLNLYERA
QHITGNLAAVADGSMNIPESGNGVADILDEARWQMEFMLAMQVPQGQAKAGMAHHKIHDVGWTGLPLAPHEDPQQ
RALVPPSTAATLNLAATAAQAARIWKDIDAGFAALCLTAAERAWNAAQANPNDIYSGNYDNGGGGYGDRFVADEF
YWAAAELYITTGDSRYLPTINNYTLERTDFGWPDTELLGVMSLAVVPATHTNSLRIAARNHIQTIASTHLTTQSA
SGYPAPLSSLEYYWGSNSVIANKLVLMGLAYDFSGNQNFALGVSKGINYLFGSNVLSTSFITGLGTNTVAQPHHR
FWAGALNSNYPWAPPGALSGGPNAGLEDSLSASRLSGCTSRPATCWLDSIDAWSTNEITINWNAPLAWVLGFYND
FAATQGGSSSSSSSSSSSVPVSSSSSSSIIPSSSSSSIQPSSSSSSMPSSSSSSSSVVASSSSSVSGGLRCNWYG
TLYPLCVTTQSGWGWENSQSCISASTCSAQPAPYGIVGAASSSSQAANRSPTLQLSANATGFEGGSMVCCTLHIN
GAASDPDGDNLTYSWQVISGNTVVASGSSSSASIHVSNQRGYEVSMTVSDGRGGVATETTFVSVYFSDYFPGSSS
SASNINSSSSSSSSSSSSAIVSSSSSVVSSSSSSAASGGNCQYVVTNQWNNGFTAVIRVRNNGSSAINGWSVNWS
YSDGSRITNSWNANVTGNNPYAASALGWNANIQPGQTAEFGFQGTKGAGSAQVPAVTGSVCQ

>Bacillus licheniformis DSM 13 (AAU40775)

MKQKAFLKMKALCLALLVIFSMSIASFSEKTRAASAEEYPHNYAELLQKSLLFYEAQRSGRLPENSRLNWRGDSG
LEDGKDVGLDLTGGWYDAGDHVKFGLPMAYSAAILSWSVYEYRDAYKESGQLDAALDNIKWATDYFLKAHTAPYE
LWGQVGNGALDHAWWGPAEVMPMKRPAYKIDAGCPGSDLAGGTAAALASASIIFKPTDSSYSEKLLAHAKQLYDF
ADRYRGKYSDCITDAQQYYNSWSGYKDELTWGAVWLYLATEEQQYLDKALASVSDWGDPANWPYRWTLSWDDVTY
GAQLLLARLTNDSRFVKSVERNLDYWSTGYSHNGSIERITYTPGGLAWLEQWGSLRYASNAAFLAFVYSDWVDTE
KAKRYRDFAVRQTEYMLGDNPQQRSFVVGYGKNPPKHPHHRTAHGSWANQMNVPENHRHTLYGALVGGPGRDDSY
RDDITDYASNEVAIDYNAAFTGNVAKMFQLFGKGHVPLPDFPEKETPEDEYFAEASINSSGNSYTEIRAQLNNRS
GWPAKKTDQLSFRYYVDLTEAVEAGYSAEDIKVTAGYNEGASVSELKPHDASKHIYYTEVSFSGVLIYPGGQSAH
KKEVQFRLSAPDGTSFWNPENDHSYQGLSHALLKTRYIPVYDDGRLVFGHEPGY

GH family 10

>Arcticibacterium luteifluviistationis (WP_111373332)

MKFKLIVLFALLVSSQTFAQYEGIENDINTNRKGELIIKAKPGAKVTVEQQSHEFWFGGAISNGLAGDYWSAEDK
KQYKEKFLENFNSAVTENAVKWGSMERKQGEVNYATVDGILEWADANEIRIRGHNIFWGIEKFVQPWLKEMDDKE
LEATLKNRAYDVAERYKGRFVEYDLNNEMIHGNFYEDRLGDGITKKMTDWFHEKDPNAKLWLNDYDILTGKRLDD
YMAQIRKFLSQGVPIAGIGVQGHLHAETFDRSELKRSLDSLSTFGLPIRITEFNTPGQRSKWVDNKTDKLTPEQE
LVKAQEIVDYYKICFAHPLVTGILQWGFWEGSNWIPASSMYRKDWSPTPAADAYQNLIYKQWWTKTSGVMATDGQ
SKVPAFYGEYKIVVDGVEKVVYLSKEKGRVEVEF

>Bacteroidetes bacterium GWB2_41_8 (OFX60056)

MKLITFLTITNLLMLQLSPVTAQITEIDQSIVANRKGEITIKSKPNAKVSVEQISHEFWFGCAIPNSFVDGSMSE
NDKKQFEEKFLKNFNSAVTENAVKWPSMERQKGQVDYSVVDGILNWTKANNIPLRAHNLFWGIPQFVQPWVKEMP
DKEVEQTLKNRAETVTARYKGQFVEYDLNNEMVHGNYYEDRLGADITKKMAEWAQSGDPDIKLYLNDYDILTGVK
LPEYMAQIRMFLKQGVPVAGIGVQGHLHAETFDRGQLKNALDSLAKFKLPIRVTEFNMPGQRSKYYNDRNLVMTP
EEEEIKAKEMVDYYKICFAHPAVEGILMWGFWEGANWIKPSSLFKKDWTPTPAAIAYQNLILKEWWTSEKGKTNR
KGELSVPAFYGKYKVTVNGKTKEIDLTRSAGKVVVKF

GH family 12

>Streptomyces halstedii (AAC45429)

MRALPHGPRTPRSLLGALLLALATVAAVLAAAPAAHADTLVCEQYGSTTIQGRYVVQNNRWGASAPQCVTATDSG
FRVTQADGAVPTNGAPKSYPSVFNGCHYTNCSPGTNLPAQVSGIASAPSSISYGFVGSAVYNASYDIWLDPTPKK
NGVNRTEIMIWLNKVGPIQPIGSQAGTASVGGRTWQVWRGSNGSNDVISFVAPSAVASWSFDVMDFVRNTIARGM
AQNNWYLTSVQAGFEPWQNGAGLAVNSFSSTVNLGTPGTGSPGAPGEPVTACEVAYATNVWSDGFTADVTVANTG
STPVDSWRLSFTLPSGQRVTNAWNATVSGPTGAVTATGLAHSAQVAAGASQTFGFQGTYSGTFSKPSGFSLNGAR
CA

>Streptomyces lividans (AAB71950)

MRTLRPQARAPRGLLAALGAVLAAFALVSSLVTAAAPAQADTTICEPFGTTTIQGRYVVQNNRWGSTAPQCVTAT
DTGFRVTQADGSAPTNGAPKSYPSVFNGCHYTNCSPGTDLPVRLDTVSAAPSSISYGFVDGAVYNASYDIWLDPT
ARTDGVNQTEIMIWFNRVGPIQPIGSPVGTASVGGRTWEVWSGGNGSNDVLSFVAPSAISGWSFDVMDFVRATVA
RGLAENDWYLTSVQAGFEPWQNGAGLAVNSFSSTVETGTPGGTDPGDPGGPSACAVSYGTNVWQDGFTADVTVTN
TGTAPVDGWQLAFTLPSGQRITNAWNASLTPSSGSVTATGASHNARIAPGGSLSFGFQGTYGGAFAEPTGFRLNG
TACTTV

>Thermotoga maritima MSB8 (AAD36592)

MRWAVLLMVVFSALLFSSEVVLTSVGATDISFNGFPVTMELNFWNVKSYEGETWLKFDGEKVEFYADLYNIVLQN
PDSWVHGYPEIYYGYKPWAGHNSGVEFLPVKVKDLPDFYVTLDYSIWYENNLPINLAMETWITRSPDQTSVSSGD
AEIMVWFYNNVLMPGGQKVDEFTTTVEINGVKQETKWDVYFAPWGWDYLAFRLTTPMKEGKVKINVKDFVQKAAE
VVKKHSTRIDNFEELYFCVWEIGTEFGDPNTTAAKFGWTFRDFSVEVVK

>Trichoderma koningii (AAM77712)

MKLIHVLPALIPAALAQTSCDQYAVFTGSDYTVSNNLWGQSAGSGFGCVTAESLSGSASWHADWQWSGGQNNVKS
YQNSQIPIPQKRTVNSISSMPTTASWSYTGSDIRANVAYDLFTAANPNHVTYSGDYELMIWLGRYGDIGPIGSSQ
GTVNVGGQSWTLYYGYNGAMQVYSFVAQTNTTSYSGDVKNFFNYLRDNKGYNAAGQYVLSYQFGTEPFTGSGTLN
VASWTASIN

GH family 44

>Paenibacillus lautus (AAA22303)

MKTRQRKRLFVSAALAVSLTMTVPMPASVNAAASDVTFTINTQSERAAISPNIYGTNQDLSGTENWSSRRLGGNR
LTGYNWENNASSAGRDWLHYSDDFLCGNGGVPDTDCDKPGAVVTAFHDKSLENGAYSIVTLQMAGYVSRDKNGPV
DESETAPSPRWDKVEFAKNAPFSLQPHLNDGQVYMDEEVNFLVNRYGNASTSTGIKAYSLDNEPALWSETHPRIH
PEQLQAAELVAKSIDLSKAVKNVDPHAEIFGPALYGFGAYLSLQDAPGWPSLQGNYSWFIDYYLDQMKNAHTQNG
KRLLDVLDVHWYPEAQGGGQRIVFGGAGNIDTQKARVQAPRSLWDPAYQEDSWIGTWFSSYLPLIPKLQSSIQTY
YPGTKLAITESSYGGDNHISGGIATADALGIFGKYGVYAANYWQTEDNTDYTSAAYKLYRNYDGNKSGFGSIKVD
AATSDTENSSVYASVTDEENSELHLIVLNKNFDDPINATFQLSGDKTYTSGRVWGFDQTGSDITEQAAITNINNN
QFTYTLPPLSAYHIVLKADSTEPVNSDLVVQYKDGDRNNATDNQIKPHFNIQNKGTSPVDLSSLTLRYYFTKDSS
AAMNGWIDWAKLGGSNIQISFGNHNGADSDTYAELGFSSGAGSIAEGGQSGEIQLRMSKADWSNFNEANDYSFDG
AKTAYIDWDRVTLYQDGQLVWGIEP

>Ruminococcus flavefaciens 17 (CAC83072)

MKKTAAFIAACVVSGCTMTAPVNGLPRATVNAAGGYDMNVTVDLKGEKKAISPLIYGVNQYTTDLRDVKTTAVRQ
GGNRMTAYNWETNASNAGSDWKHSSDNNLSDSDDPADCVQVLSKQAAKYNVNYKLTTLQLAGYVSADKNGPVSEA

EKAPSDRWNKVVLTKNAPFADTPDLTDGVVYMDEYVNYIINKLGDSQSAEGIQGYSLDNEPVLWNDTHSRMHPDP
VTIEELGSKSVEMAKAVKKLDPKAEVFGPALYGYTAFDHLDDDDAHTEWEEIKKANNYHWYLDCYLDHMHKASEE
NGARLLDVLDIHYYSESARKGIEDRLQSVRTLYEPGFSENSWIGQWCMENVPILPTIQKSIDTYYPGTKLGISEY
NFGGGDDASGTIAQAEALGCYADQGVYFASLWGGEPFILSGIQLYTNYDGKGGCFGDTLIPASTGDVSKSSTYAA
VNAKDDSKVTVMVTNKDLKENENAVIDLRNADKSYKSAAVYAVFGDSEEIRLIDIIKDVKDNKVKTELPAFSAAM
VVVSDQADAFDGLKTYEETKTETKTVEFTDIESMTNDKGFVVVPIEDAEHLSKIIINGAVTSSAGSSWATAGCAV
CMNVTAKDGSGFWTYKSYNLPLGSKSSATVKFDGIFTKETGEGADKVKEDLEATVAEGKVELQKWWDASEKSEES
SEDKIEVKYSSIQVVYEYAQGEAPKVTTTTTSSAVTTTTTSVATTTSSTTAPDKDVVYGDANCDGNVDLADAILI
MQSLANPNKFGLNGSAEKHLTEKGKRNADCCDPGSGLTNDDALSIQRLLLHLIDKLPE

>Uncultured microorganism (AGZ87466)

MGLALDAAGSLYVANCGDYPCGNSNNITIFDAPATQTTATATATPTATTANVTPTPTTTTSTVTATPTATATRTP
TPTATSTQVTPTTADISLSVDLSTNRHPISDAIYGIHYVEDEAFAAEIDLPVRRWGGNDTTRYNWKNSMFGNADW
YFENEYKPTSADDFIAQNKRTGADSIITLPMSGWMTKDPGQPGNSATYPCSFDTRKYNYTPQALPNGLPSYDTAD
PKRSHCGSGVTRYENGRAVYFQGNDPTDTSFAIDSSWTSEWIAHLVQTFGAADKNGVRFYGLDNEPDLWHETHRD
IFPIALTYDQIRQRAYDYAAVVKAADPAAQILGPVLMGWTYYWHSPRDGQQELWTTRPDRMSHGDVPLVPWYLQQ
MAEYEAQNGIRLLDYLDLHFYPQNGVDLRDAGDANLQALRLRSTRALWDPTYVDESWIADAGPDGGVVQLIPRMR
EWVTQNYPGTKLAITEYNWGALDHINGALTQADILGIFGREGLDLATLFDTPYGNGGNFSPQSPAAYAFRLFRNY
NGAGAKFGDISVEAVSSDQEKLAVYAAERSSDNALTLLVINKSSSPLTADLSIINRPSSLSTMGQIYRYSAGAPN
TIVTAADLSLAGDSTQTTFPASSITLIVVQSSPTDLPHRLYLSALNRH

GH family 45

>Cellvibrio japonicus Ueda107 (ACE82688)

MNLLSGWVRPLMLGCGLLGAALSAGSIQAAVCEYRVTNEWGSGFTASIRITNNGSSTINGWSVSWNYTDGSRVTS
SWNAGLSGANPYSATPVGWNTSIPIGSSVEFGVQGNNGSSRAQVPAVTGAICGGQGSSAPSSVASSSSSSSVVSS
TPRSSSSSVSSSVPGTSSSSSSSVLTGAQACNWYGTLTPLCNNTSNGWGYEDGRSCVARTTCSAQPAPYGIVSTS
SSTPLSSSSSSRSSVASSSSLSSATSSSASSVSSVPPIDGGCNGYATRYWDCCKPHCGWSANVPSLVSPLQSCSA
NNTRLSDVSVGSSCDGGGGYMCWDKIPFAVSPTLAYGYAATSSGDVCGRCYQLQFTGSSYNAPGDPGSAALAGKT
MIVQATNIGYDVSGGQFDILVPGGGVGAFNACSAQWGVSNAELGAQYGGFLAACKQQLGYNASLSQYKSCVLNRC
DSVFGSRGLTQLQQGCTWFAEWFEAADNPSLKYKEVPCPAELTTRSGMNRSILNDIRNTCP

>Komagataella phaffii GS115 (CAY71902)

MSTLTLLAVLLSLQNSALAAQAETASLYHQCGGANWEGATQCISGAYCQSQNPYYYQCVATSWGYYTNTSISSTA
TLPSSSTTVSPTSSVVPTGLVSPLYGQCGGQNWNGATSCAQGSYCKYMNNYYFQCVPEADGNPAEISTFSENGEI
IVTAIEAPTWAQCGGHGYYGPTKCQVGTSCRELNAWYYQCIPDDHTDASTTTLDPTSSFVSTTSLSTLPASSETT
IVTPTSIAAEQVPLWGQCGGIGYTGSTICEQGSCVYLNDWYYQCLISDQGTASTTSATTSITSFNVSSSSETTVI
APTSISTEDVPLWGQCGGIGYTGSTTCSQGSCVYLNDWYFQCLPEEETTSSTSSSSSSSSSTSSASSTSSTSST
SSTSSTSSSTSSSSIPTSTSSSGDFETIPNGFSGTGRTTRYWDCCKPSCSWPGKSNSVTGPVRSCGVSGNVLDAN
AQSGCIGGEAFTCDEQQPWSINDDLAYGFAAASLAGGSEDSSCCTCMKLTFTSSSIAGKTMIVQLTNTGADLGSN
HFDIALPGGGLGIFTEGCSSQFGSGYQWGNQYGGISSLAECDGLPSELQPGCQFRFGWFENADNPSVEFEQVSCP
PEITSITGCARTDE

>Rhizophlyctis rosea (AWL24828)

MLAKSVIVSSLLLAPGALAASAYGQCGGKGWTGDTTCPSDYVCVASGEYYSQCVLGTNGGSNGSGSGQATTTTTT
TSNRPSTTTVTTTRPPSAATTPGNTTRYWDCCKPSCSWPGKGGSGPAKSCAKDGVTALGSNTMNSCDGGSAYTCN
SYQPIVVNDNLSYGFAAAKIAGLSESDWCCACYELNFTTGPVAGKQMIVQVLNTGGDLGPGHFDLQIPGGGVGLF
NGCQSQWGAPGSGWGAQYGGISSASDCSQLPAQLQSGCNWRFGWFKNADNPLVNFRKVTCPSELTGASGCSHA

>Ampullaria crossean (ABR92638)

MKLFYLLCLAVPVLEAAQLCQPDAHGVRRFNGRPCASTTRYVDGHKGACGCGQKGSDTPFPWNLQKHVTAPSERY
FDDGGSNLWCGKNCGKCVRLTPTGGFVPGKGGAPPNHNPVVFMVTNACPINGNEEWCGISGKPGTNHVNSHGYEV
HFDLQDQVGQVEALHWDNPEVTWEEVPCPGDLQANYQQCECHNSD

>Cryptopygus antarcticus (ACV50414)

MKVFVVLAAIVAIANGLTSGSGVTTRYWDCCKPSCSWGGKASVTKPVRTCKANGNTTIDSNTQSGCNGGSSYVCN
DQQPFTQGNVGYGFAAASISGQPESQTCCACYEMTFTNTAISGQKMIVQVTNTGSDLNGNHFDLMIPGGGVGIFN
GCQSQWGAPSNGWGQRYGGISSQSECNQLPTSLRAGCNWRFGWFKNADNPSMKFTQVRCPTILTQKSQCVRTPGP

GH family 48

>Bacillus licheniformis DSM 13 (AAU40776)

MDNKTRFMQLYEQIKNPNNGYFSPEGIPYHSVETLICEAPDYGHMTTSEAYSYWLWLEAMYGRYTQDWSKLEAAW
DNMEKYIIPVNEGDGNEEQPTMNYYNPSSPATYAAEHPYPDLYPSALTGQYPAGNDPLDAELKATYGSNETYLMH
WLLDVDNWYGFGNLLNPSHTAVYVNTYQRGEQESVWETVPHPSQDNQTFGKPNEGFMSLFTKENQAPAPQWRYTN
ATDADARAVQAMFWARQWGYSNTNYLEKAKKMGDFLRYGMYDKYFQEIGSAADGSPSRGAGKNACHYLMAWYTAW
GGGLGQYANWAWRIGASHVHQGYQNPVASYALSTAEGGLIPNSSTARSDWEKALKRQLELYTWLLSSEGAVAGGA
TNSWNGNYSAYPQNVSTFYEMAYTEAPVYHDPPSNNWFGMQVWPLERVAELYYIFAEKGDKSSESFHMAKHVIEK
WIAYSLDYVFVGERPVTDEEGYYLNDAGERVLGGQNPQIAVQSDPGEFWIPANLEWSGQPDPWKGFDSFTGNPGL
HVTTKNPSQDVGVLGSYIKTLVFFAAGTKAETGGFTALGNKAKNLAKELLDAAWSKNDGIGIAAEEEHEDYIRYF
TKEIYFPNGWSGRNGQGNTIPGPNTVPSDPAKGGNGVYISHAELRPKIKNDPMWPYLENKYQTSWNPNTGKWENG
LPTFVYHRFWSQVDMATAYAEYDRLIGNA

>Acetivibrio thermocellus ATCC 27405 (ABN51312)

MKLRYKVRRRRRIITCCGIIAAVIVVSTLIITIKNSFKPSRQSSNSNVTYSKENVQSVYSDRFIALFEDIQKQGY
LSEEGIPYHSIETLLVEAPDYGHLTTSEAMSYMVWLGATYGKLTGDWTYFKDAWDKTEQYIIPDPERDQPGVNSY
IPTQPAQYAPEADSPEKYPTPGDINAPTGIDPIADELASTYGTKAIYQMHWLLDVDNWYGYGNHGDGTSRCSYIN
TYQRGSGESVWETIPHPSWEDFRWGQVNNGGFLKLFGNFGEPVRQWRYTSASDADARQIQATYWAYLWSKEQGKE
KELQPYFEKAAKMGDYLRYTFFDKYFRPIGVQDSGRAGTGYDSCHYLLSWYASWGGDINGTWSWRIGSSHCHQGY
QNPMAAYALAKESIFTPKSKNAKKDWEQSLDRQIELFLYLQSAEGAIAGGVTNSWSGAYGKYPEGTSTFYDMAYD
PHPVYNDPPSNRWFGFQAWSMERIMEYYYLTGDSRVKELCKKWVSWAIENTRLKSDGTYEIPSTLEWSGQPDPWT
GKPSENKNLHCTVTEWTVDVGVTASYAKALIYYAAATEKHEKKIDDKARETAKQLLDRMWHNYRDKKGVAAKEPR
ADYKRFFDEVYIPHDFSGINAQGAEIKNGITFIDLRPKYKEDKDYKMVEEAIKSGKDPVMTYHRYWAQAEVAMAN
AMYHIFFEQKKDGLVPGINSDDSNSSSETQKSETPDYTPDSLSTSDATITESPANTNSPDENPSPQNTSAPTNVP
LNPANTPYNSSNAAPNSPNTPARPSNTTAGSPAGNNTVGRLILQYANGNGSDTTNTINPRFKLINNSGSPVKLSD
VKIRYYYTIDGEKGQQFWCDWSSAGNSNVTGKFVKLSSPKNNADYYLEIGFTEGAGSIEPGMSVEVQARFSKDDW
SNYSQANDYSFSASANDYGNSNHIALYISGRLVSGNEP

>Ruminiclostridium cellulolyticum H10 (ACL75108)

MSKNFKRVGAVAVAAAMSLSIMATTSINAASSPANKVYQDRFESMYSKIKDPANGYFSEQGIPYHSIETLMVEAP
DYGHVTTSEAMSYYMWLEAMHGRFSGDFTGFDKSWSVTEQYLIPTEKDQPNTSMSRYDANKPATYAPEFQDPSKY
PSPLDTSQPVGRDPINSQLTSAYGTSMLYGMHWILDVDNWYGFGARADGTSKPSYINTFQRGEQESTWETIPQPC
WDEHKFGGQYGFLDLFTKDTGTPAKQFKYTNAPDADARAVQATYWADQWAKEQGKSVSTSVGKATKMGDYLRYSF
FDKYFRKIGQPSQAGTGYDAAHYLLSWYYAWGGGIDSTWSWIIGSSHNHFGYQNPFAAWVLSTDANFKPKSSNGA
SDWAKSLDRQLEFYQWLQSAEGAIAGGATNSWNGRYEAVPSGTSTFYGMGYVENPVYADPGSNTWFGMQVWSMQR
VAELYYKTGDARAKKLLDKWAKWINGEIKFNADGTFQIPSTIDWEGQPDTWNPTQGYTGNANLHVKVVNYGTDLG

CASSLANTLTYYAAKSGDETSRQNAQKLLDAMWNNYSDSKGISTVEQRGDYHRFLDQEVFVPAGWTGKMPNGDVI
KSGVKFIDIRSKYKQDPEWQTMVAALQAGQVPTQRLHRFWAQSEFAVANGVYAILFPDQGPEKLLGDVNGDETVD
AIDLAILKKYLLNSSTTINTANADMNSDNAIDAIDYALLKKALLSIQ

GH family 51

>Fibrobacter succinogenes (AAC45377)

MIKQSLKVASLAVLGLSVTAAMAQPKRPHLAVYKFFDEQYRPGGYDYSYGGTSKGVTITKSGGYKSKAALNIKLD
PKEYSGASICLYNEFFDLNKYMLDSKVEFMIKGKNGGESVKVGLLDEEVSDGKKTQVVLPMNKYIEGGAVTTDWK
KVSIPLVDFPDRGLYWDNTRKSEFPSRIDWDKIAEIRFSIDKSAASEFEVWVDNIEIVKGNKKAAPKKQMVYWDE
NNDIIDGPKNPEKLDGKAKTLATFYDNQVKGFSYSYGGLTAQREAQSKTPGNKNVLAMYIDNNDWSGVTYSLGEG
KFIDLSKVRDKGGLYFWIKGKLGGEKLYVGILDNQGNDIKSQTKVGLNDWIKVSKDWQLAKIPLKRFTDKGKAWD
ANKSAEVAKDIKWDKIQEIRFSVGKGENAGEPGKPAPVTVFVDQITFTSNIDWVDPDLKWDSFKSNAPDYVISDF
EGKFAKDKWEPSTGPKSQLKFKVENCAEFKSNCLNIEHYLLADWVDVVLDMQKNGRPAADRDWTKHWGIMFDVYS
EKAWQSITVQVQDAGNEIFVSNVGAPKGKTTILVPFRTFGKFPYYQPPNAVENGLFDLKGVTALDFKPSGEGTAG
GFKVDNIRLTNQREVKAKERPAVIKVLVKGEKDVLNPNISGGLFGINAALWDGDMLDNKNFKVQTREYAKRINHG
IIRYPGGLRADDDHWKEILDNHDWMVDTDEFLEWLKKTGSNAMFTVNFGSGTVKEAADWVKHTNIDKKAGILYWE
IGNEIYGNWHPYYEKYGKDGGTIYGKRARKFIEAMKKVDPTIKVAVLGVLEGDWNDKVLAETGDIADGLIVHHYP
QHFGEENDFAMLSAPQTLTAIYERLHKVVDKWTKKYNKDKKIELWLTEWNSVDFNPGPQTLSVENGLFVADYLGM
LATENVDNAQYWDIHNDITPEGGDYGYLTRSGEECMNCPRPSYWAFQMASDALRGKLMKTTIKGDEDALLTAYWT
VNGNKKQLLLVNKSPYSEFDIKLDIPGFKGKAKVQTLDKTSEKLKEGWANDPSKKAKTVDISKGIKVGKRTLTLI
TLE

>Alicyclobacillus vulcanalis (CEH24710)

MSVHSAATHAKAHVGVRAADMAAASMSAEIQILHDALTASELSSVQAAAQAAANLPASTWVSWLYPSASSPSAAQ
TQTAQALGALLTLVTYGAVADDGQNIAQNLQTLQSTSPLLSPAAVSMFYQNFFVLVGQSSKSVLSGQATTSTAGH
ALAQAAALTPQLAAYLRQSGLSPDDLARAYVSFASAVDSQGAAQTALLTRICTNILGFGAPTSTATITVNAAANL
GQVPTTAFGLNAAVWDSGLNSQTVISEVQALHPALIRWPGGSISDVYNWETNTRNDGGYVNPDDTFDHFMQFVNA
VGSTPIITVNYGTGTPQLAADWVKYADVTHHDNVMYWEIGNEIYGNGYYNGNGWEADDHAVAGQPQKGNPGLSPQ
AYAQNALQFIKAMRAEDPSIKIGAVLTMPYNWPWGATVNGNDDWNTVVLKALGPYIDFVDVHWYPETPGQETDAG
LLADTDQIPAMVAELKREVNTYAGSNAKNIQIFVTETNSVSYNPGEQSTNLPEALFLADDLTGFIQAGAANVDWW
DLFNGAEDNYTSPSLYGQNLFGDYGLLSSGQTTQNGWQEPPANTPLPPYNGFQLVSDFAQPGDTMLGSTTSQSAI
DVHAVRKPNGDISLMLVNRSPSAIYSANLNVLGFGPFVVTHALAYGEGSSRVAPMPVLPVPGAPIKLMPYSGIDL
TLHPLIPAPHAAAQVTDTLTLSSPTVTAGGAETLSASFQADRPVHHATVELELYDSTNDLVATHTVSDVDLQPGS
ATSETWSFTAPAANGNYRVEAFVFDPVTGATYDADTQGAVLTVNQPPQATYGDIVTKDTVITVNGTTYDVPAPDA
GGHYPSGTNISVAPGDTVTVQTTFVNVSSTDALQNGLIDMEVDGSNGAILQKYWPSTTLLPGQSETVTATWQVPA
NVAAGTYPLNFQAFNTSSWTGNCYFTNGGVVNFVIS

>Uncultured bacterium (CAF22222)

MNENRVGDGNPVILYVYDVKFIEEIPGYVCPEEFWGAFESDAPDLQLHDFATEQDRNWETSTGPKSSGSFEFVEA
APEESEGYSIKISYSLHDWVDIIYNYRDNNSPAEHRDWTNHWGLKFDVYTERAFQPFNVQVNDSGNELWIATAGA
EKGWTEVIVAFRDFYKFPHWQPPDAVHTGNFDIENVIALDFKPSGEGTSAQFKVHNVRLTNDREARRAPVPEYVA
VNVTGSFDEVVTEQINEGIFGINAALWDGDLLLPETVEYVKAVNHHVLRYPGGLRADDDNWKEVLAAQDWMVDTD
QFLEFCRATNTEAMITVNFGSGTVEDAADWVRHHNVENDDNVRYWEVGNELYGDWHPFQTTAEDYGKRTREFIIA
MKEVDPTIKVTFVGVLDGEWNRIVLEHVKDVADGINVHHYPQTTGEENDAGLLSSPQTLDDIIPSVRRQLAEWGE
PGREYEIWLTEWNSVDFEPGPQTLSIVNGLFVADYLGMLAKHNIEQASYWDIHNDITDQGGDYGYLSRTGAPDGC
NVPRPSYWAFKMASHSLGRGSLLNSGTESDYVTSYLTYDNGKKSLMLVNKYPQTRADVTINIPGFEGRGTKQVLT
AESGQPGPEKGNFRVRQGTELALPPYSITTITLD

GH family 74

>Thermotoga maritima MSB8 (AAD35393)

MLRSFLILFLAILGVVFGATFEWKSVEINGGGFVPGIIFHPASPGLLYARTDVGGLYRWDEETKRWKQLFDFLRR
DQSDYMGVLSVALDPSDPKRIYAMTGKYTQDWAGYGAILISEDYGETWTIVNLDKYGIKVGGNEDGRNAGERLQV
DPNFSSVLFMGTTKYGLWKSEDFGKNWKKVDSFPSTSVTFVLFDEKSGEKGSPTPRIFVGCSEPKGIFVTEDGGT
TWNVLPNLPNDLIPLRGKIHDGILYVTLSNALGPNGATRGAVMKYVIADQKWYDVTPMKGDFGYCGIDVQENVVI
VSTLDRWYPHDEIFISLNGGETWRPLLEKANFDINKAPWIKDLNPHWISDVKIDPFDMNRAIFTTGYGVWVTYEL
KKSFEGMGKPVKWIFENRGLEETVVLQLVPPIGERPLLSAIADWGGFRHESLDTPPSSMYKPLKWTSLGIAFAYQ
NSKFVARVHTYTYPFLSYSEDGGINWREIETVPEGITDGGRLSLAVSNDGKTLVWSPANHEVIVSSDKGKSWKKA
ISVPVPEFNYFPASDPVNPSKFYIFDWKNGDFLISKDGGKSFMKGAKLPSFDNWWVSLYSFPVLAPDREGDIWLA
LQWNGLYRSKDGGITFERLGNVDIAYVIGFGAPKPGTDYPAIYLNGMVNGVYGIFMSTDEGKTWMRINNDKHQFG
WIHYMIGDMNEFGRIFLGTEGRGIIVGEVKEE

>Streptomyces coelicolor A3(2) (CAA20642)

MRRTRILTVLLALAAGLLAGSPPAASAAEPAPRAAVAADSYTWKNARIDGGGFVPGIVFNRTEKDLAYARTDIGG
AYRWQEESHTWTPLLDHVGWDDWGHTGVVALASDAVDPDRVYAAVGTYTNDWDPTNGAVLRSADRGASWEKADLP
FKLGGNMPGRGMGERLAVDPHDNDVLYLGAPSGHGLWRSTDAGVTWSEVTAFPNPGNYAQDPNDTSGYASDNQGI
TWVTFDESTGGGAGTATRTLYVGVADKENAVYRSTDAGATWERLAGQPTGYLAHKGVLDAENGYLYLAYSDTGGP
YDGGKGRLYRYATATGTWTDISPAAEADTYYGFSGLTVDRQRPGTVMATAYSSWWPDTQIFRSTDSGATWSQAWS
YTSYPDRENRYTMDVSSSPWLTWGANPAPPEQTPKLGWMTEALEIDPFDSDRMMYGTGATVYGTENLTNWDDEGG
TFAVEPMVRGLEETAVNDLASPPSGAPLLSALGDVGGFRHTSLTEVPSMMYTSPNFTSTTSLDFAETKPDVVVRA
GNLDSGPHIAFSTDNGANWFGGTDPSGVSGGGTVAAGADGSRFVWSPEGAGVQYTTGFGTSWQASTGLPAGAIVE
SDRVNPATFYGFKSGRFYVSTDGGATFTASAATGLPAGDGVRFKALPGGEGDVWLAGGAADGPYGLWHSTDGGGT
FTRLPGVDAADTVGFGKAAPGASYQTLFTSAEIGGVRGIFRSTDAGATWTRVNDDAHQWGWTGAAITGDPRVYGR
VYVATNGRGVIYGDTSDTGGGTDPGPGPDPTPTGACEVTYTVTNQWPGGFQADVRLTNTGTSAWNGWSLDWSFPG
GQEVTRMWNAEHTQAGTSVTARNVGWNAGVAPGASVGFGFTGSRSGTNAEPEGFAVAGRACPTAT

GH family 124

>Acetivibrio thermocellus ATCC 27405 (ABN51673)

MKRKILIVFVLLSIAISQFLYFEVGCFDVYAWNKAVIGDVNADGVVNISDYVLMKRYILRIIADFPADDDMWVGD
VNGDNVINDIDCNYLKRYLLHMIREFPKNSYNSAPTFTPIPTFTPTPTPTKAPAAPANTQSGILNDGYFPPGTSK
HELIARASSLKVSEVKAIIKKQVDEHWDVIRDVCGFKNKEVAYAFFFGMATRESTFRAATETGSGASHAFGPLQT
AETAYANANPNYMPEHNVPEMHQYDFTEYNFYDVGISVHMGIRHFLHFARLAKEKYSGRDIARHGLMGYNTGWID
GADESWIVRYADETAALGAWYLRNNHMSDDEFTWDTDPRVDRSNPWEIYY
